# Potential effect modifiers for treatment with chiropractic manipulation versus sham manipulation for recurrent headaches in children aged 7–14 years: development of and results from a secondary analysis of a randomised clinical trial

**DOI:** 10.1186/s12998-023-00492-2

**Published:** 2023-07-11

**Authors:** Susanne Lynge, Werner Vach, Kristina Boe Dissing, Lise Hestbaek

**Affiliations:** 1 Private Chiropractic Practice, Vivaldisvej 6, 9700 Broenderslev, Denmark; 2 The Chiropractic Knowledge Hub, University of Southern Denmark, Campusvej 55, 5230 Odense M, Denmark; 3 Basel Academy for Quality and Research in Medicine, Steinenring 6, 4051 Basel, Switzerland; 4 Department of Sports Science and Clinical Biomechanics, University of Southern Denmark, Campusvej 55, 5230 Odense C, Denmark

**Keywords:** Children, Headache, Chiropractic, Manual therapy, Effect modifier, Clinical trial

## Abstract

**Background:**

A recent randomized controlled trial (RCT) investigating the effect of chiropractic manipulation in 199 children aged 7–14 years with recurrent headaches demonstrated a significant reduction of number of days with headache and a better global perceived effect (GPE) in the chiropractic manipulation group compared to a sham manipulation group. However, potential modifiers for the effectiveness of chiropractic manipulation of children with recurrent headaches have never been identified. The present study is a secondary analysis of data from that RCT and will investigate potential effect modifiers for the benefit of chiropractic manipulation for children with headache.

**Methods:**

Sixteen potential effect modifiers were identified from the literature and a summary index was prespecified based on clinical experience. Relevant variables were extracted from baseline questionnaires, and outcomes were obtained by means of short text messages. The modifying effect of the candidate variables was assessed by fitting interaction models to the data of the RCT. In addition, an attempt to define a new summary index was made.

**Results:**

The prespecified index showed no modifying effect. Four single variables demonstrated a treatment effect difference of more than 1 day with headache per week between the lower and the upper end of the spectrum*: intensity of headache* (*p* = 0.122), *Frequency of headache* (*p* = 0.031), *sleep duration* (*p* = 0.243), and *Socioeconomic status* (*p* = 0.082). Five variables had a treatment effect difference of more than 0.7 points on the GPE scale between the lower and the upper end of the spectrum*: Frequency of headac*he (*p* = 0.056), *Sport activity *(*p* = 0.110), *Sleep duration *(*p* = 0.080), *History of neck pain *(*p* = 0.011), and *Headache in the family *(0.050). A new summary index could be constructed giving highest weight to *History of neck pain* and *Headache in the family* and *Frequency of headache*. The index suggests a difference of about 1 point in GPE between low and high values of the index.

**Conclusion:**

Chiropractic manipulation offers a moderate benefit for a broad spectrum of children. However, it cannot be excluded that specific headache characteristics, family factors, or a history of neck pain may modify the effect. This question must be addressed in future studies.

*Trial registration*: ClinicalTrials.gov (Albers et al in Curr Pain Headache Rep 19:3–4, 2015), identifier NCT02684916, registered 02/18/2016—retrospectively registered.

**Supplementary Information:**

The online version contains supplementary material available at 10.1186/s12998-023-00492-2.

## Background

There has been an increasing prevalence of headache in school children during the last decade [1] and there is an increasing body of research investigating treatment of pediatric headache. Nevertheless, investigations into pharmacological interventions have still not demonstrated sufficient effective and safe treatment options [2, 3] and the question of why effects and side effects of drug treatment are so variable remains [4]. With regard to non-pharmacological interventions for headache, the evidence is highly heterogenous [5] making it difficult to determine when to initiate the various options of treatment, in which combination it should be administered, and who may benefit the most.

Spinal manipulation is a common non-pharmacological treatment for headache in adults [6]. Strong evidence is lacking, but there is some evidence in favor of manipulation for treatment of headaches in adults [6–9], and the Danish National Board of Health recommends consideration of manipulation in the treatment of headache [10]. Also children receive treatment in form of spinal manipulation, and in Denmark headache is the second most common complaint from children seeking chiropractic treatment [11]. Nevertheless, knowledge about the effectiveness of spinal manipulation on pediatric headache is very limited.

A recent randomized controlled trial (RCT) investigating the effect of chiropractic manipulation in 199 children aged 7–14 years with recurrent headaches demonstrated a significant reduction of number of days with headache and a better global perceived effect (GPE) in the chiropractic manipulation group compared to a sham manipulation group [12]. However, potential modifiers for the effectiveness of chiropractic manipulation of children with recurrent headaches have never been identified. The identification of patient characteristics that may influence the outcome of treatment, either positively or negatively, in the pediatric population suffering from headache is essential to enhance clinical decisions of treatment in the future [13].

## Methods

### Aim

The present study is a secondary analysis of data from the RCT mentioned above. We aim to investigate a series of patient characteristics measured at baseline with respect to their potential to increase or decrease the benefit of chiropractic manipulation for children with headache. In addition, two attempts are made to summarize the information from these variables into a simple index.

### Design

This study is partly confirmative with respect to validation of a series of candidate variables which are identified based on the literature and the personal experience of the PI. The study is partly exploratory with respect to developing a data-driven suggestion of an index summarizing the information from all candidate variables.

### Setting and participants

The RCT was conducted in two clinics in Northern Denmark between November 2015 and April 2020. Invitations were sent through the Danish School Information Network, local newspapers, television, social media, and radio. Screening and treatments were administered by the investigating chiropractor with 34 years of experience in paediatric private practice. Children aged 7–14 years of age were invited to participate if they had suffered from headache for at least half a year with a minimum of one episode of headache per week, and in addition the investigating chiropractor had to be able to identify at least one musculoskeletal dysfunction in the spine, pelvis and/or temporomandibular joint. Exclusion criteria were contraindications to spinal manipulation, red flags requiring referral to other types of health care at the initial screening visit, other treatments for headache within the past three months, or failure to report pre-randomization baseline data.

The recruitment process included a detailed baseline questionnaire prior to start of a four-week pre-treatment phase. The translated version of this questionnaire is provided in Additional file 1: A.

### Intervention

There were two groups randomized with 1:1 allocation using random block size administered by a data manager at the Chiropractic Knowledge Hub. Participation period was 4 months in both groups. Parents and children were blinded for group allocation. All parents and children were given written and oral advice on general lifestyle generally believed to be beneficial to reduce headache. This regarded regular meals, enough liquid and sleep, reduction of screen time and at least half an hour of physical activity per day. This information was given before allocation.

The intervention group received chiropractic manipulation, a high-velocity, low-amplitude thrust resulting in an audible cavitation directed at specific, individually identified, dysfunctions of one or more joint(s) in the spine, pelvis and/or temporomandibular joint. All treatments were modified to fit age, size, and the individually identified dysfunctions of each child, as were the number of treatments [12].

The control group received sham manipulation treatment, where a patient placement similar to the one used in the intervention group was used, but in this group only gentle pushes with a broad, non-specific contact away from the spinal column were given with no resulting cavitation. This method followed a previously validated protocol by Chaibi et al. [14]. The children in this group should receive approximately eight treatments during the four months participation period.

More details can be found in the published protocol [15].

### Outcomes

In the RCT, four primary outcomes were considered. Three were based on weekly text message (SMS) reports from the participating children and their parents: The frequency of headaches (Number of days with headache per week), the headache intensity on a numerical rating scale (NRS) from 0 to 10, and the number of headache pills per week. A 4-weeks pre-treatment period was compared with the final 4 weeks of the study period (week 14–17) at the individual level by computing change scores. The fourth primary outcome was the global perceived effect (GPE) after 4 months, based on a final SMS.

Due to reporting issues with respect to the number of pills, this variable could only be analyzed at the level of yes/no per week, and no difference between the intervention groups was observed. Also, for headache intensity, no difference was found. In contrast, a significant difference was found with respect to the change in the frequency and the global perceived effect. The average change in number of days with headache from baseline was − 0.813 in the chiropractic manipulation group and − 0.406 in the sham manipulation group, i.e., chiropractic manipulation decreased the number of days with headache on average by 0.41. The GPE was assessed on a 7-point scale with low values indicating a favorable outcome. The average numbers were 2.62 and 3.24 for the chiropractic manipulation group and the sham group respectively, i.e., the chiropractic manipulation improved the GPE on average by 0.62 [12]. These two outcomes were included in the present secondary analysis.

### Overall analytical strategy

A series of potential treatment effect modifiers was identified based on the existing literature on headache characteristics and risk factors for headache in children. In addition, an expected benefit index was created based on most and least favourable conditions for a benefit from chiropractic treatment. These conditions reflected the expectations of the principal investigator (SL), based on her clinical experience.

For the confirmative part, the identified potential effect modifiers led to a series of candidate variables based on the baseline data available. The expected benefit index was also considered as a candidate variable. The modifying effect of each candidate variable was examined by considering the difference in the primary outcomes between the two intervention groups, stratified by the values of the candidate variables, and assessing the statistical significance of this association.

In the exploratory part, an attempt was made to construct a new index variable by combining all single candidate variables The potential value of this new index was depicted in the same manner as for the candidate variables, except for the statistical significance of the association, which could not be assessed.

### Selection of potential treatment effect modifiers for the effectiveness of chiropractic manipulation

Since the RCT, this study is based on, is the first to study the effect of chiropractic manipulation for headache in children, we cannot base the choice of potential effect modifiers on results from previous RCTs. Neither are we aware of any attempt to identify potential effect modifiers based on observational data. Therefore, as recommended by Hancock et al. [16], available baseline variables were selected if associations with risk or prognosis of headache in children had previously been demonstrated in the literature without considering theoretical explanations for modifying effects. In addition, established headache characteristics were selected.

In some cases, baseline variables were combined to describe constructs which can be aligned with the variables described in the literature. This process is described in Additional file 2: B. Table 1 presents the finally considered candidate variables, which were either headache characteristics or related to constructs identified in the literature.

**Table 1:** Description of the 16 candidate variables

Candidate variable	Formation from baseline questionnaire	
*Headache characteristics*		
Intensity of headache	Numerical rating scale (NRS) from 0 to 10	
Frequency of headache	Number of days with headache per week. 1–2 days/3–5 day/nearly every day	
Duration of headache	0.5–1 year/1–3 years/more than 3 years	
Length of episodes	< 2 h/half day/whole day/day and night	
Absence from school	0/1–5/5–20/ > 20 days last year	
Co-occurring symptoms	Summary index based on four binary items: nausea, vomiting, light sensitivity, and sound sensitivity	
Migraine-tension-type	Continuous index based on severity and co-occurring symptoms (high values indicating migraine, low values tension type headache). Derived from previous analyses of the same cohort [17]	
*Variables identified from the literature*		*Relationship with headache*
Age	Reported age in years	Headache prevalence rises with age and there is a significant increase in headache prevalence after age 12 [18, 19]
Sport activity	"0 times”,”1–3 times", or " > 3 times"	Low physical activity is associated with recurrent headaches in adolescents [20–22]
Screen time	“low”, “normal”, or “high” based on age-specific cut points for the self-reported screen time	A forward head posture, as often assumed in front of computers, has been associated with neck and shoulder pain [5, 23]
Sleep duration	“low”, “normal”, or “high” based on age-specific cut points for the self-reported sleep duration	Poor quality of sleep is associated with headache in children [24–26] and this association is particularly evident in migraine [24]. Children with headache report more daytime symptoms of sleep disturbances, including fatigue, tiredness, and sleepiness [27]. Furthermore, excessive screen time may influence headache mediated by less sleep, and reduced sleep duration can be an indicator of an unhealthy lifestyle [28]
Trauma experience	Summary index based on three items on lifetime trauma experience (no need for treatment, need for treatment, hospitalization)	The cervical spine is the most commonly injured region of the spine in young children [29, 30] and thus cervicogenic headache might be a consequence of cervical dysfunction
History of concussion	Binary item on lifetime experience of concussions	In adults, headache associated with head injury is estimated to persist for 12 to 24 months after the injury in 20–30% [31]
History of neck pain	Report of neck pain within the past year	The cervical spine is the most commonly injured region of the spine in young children [29, 30] and thus cervicogenic headache might be a consequence of cervical dysfunction
Socioeconomic status	Income is here considered as a proxy for socioeconomic status: self-reported annual income in 8 categories (labelled in 1000 €)	Socioeconomically disadvantaged children are more prone to headache [32]
Headache in the family	Presence of headache in the parents: “none”, “one parent “, or “both parents”	A history of headache in a first-degree family member has been reported in up to 72% of children with headache with a predominance of maternal headache[19, 25, 33, 34]

### Expectation benefit index

The principal investigator (SL) presented two fictive cases, representing her expectations of the highest and the lowest chance of a favorable outcome following chiropractic manipulation. These cases were based on her clinical experience after 30 years’ experience with chiropractic manipulation of children and followed three lines of arguments:

- Children may benefit most from chiropractic manipulation if the cause of the headache is of biomechanical origin with no other underlying conditions or the lifestyle and/or psychosocial environment of the child.
- Previous trauma (particularly to the neck) may have affected the spine and acted as precursors for a mechanical dysfunction where chiropractic manipulation treatment is indicated.
- Chiropractic manipulation treatment will benefit, as many other types of treatment, from a healthy lifestyle of the patient. In our context this may be an active lifestyle of the child with enough physical activity, limited screen time, and sufficient psycho-social support

The two cases were described as follows:

Case 1—most favorable outcome:

- Age 7–12 (before puberty)
- Participate in sport, average or elite, or other leisure time activities/hobbies
- Screen time not above average
- Likely to have reported at least one trauma

Case 2—least favorable outcome:

- Age 13–14
- No sport or other leisure time activity
- Screen time above average of age matched peers
- No trauma reported (maybe due to lack of physical activity)
- Daily headaches

This expectation let us define the following *Expected benefit index* with high values reflecting a better chance to benefit. This index is based on giving a half point or a full point to certain conditions:

- Age: one point if ≤ 9 and half point if ≤ 12
- Sport activity: one point if more than 0 days per week
- Screen time: one point if not above normal level
- Trauma experience: one point if at least two traumas reported or one requiring treatment, and half point if at least one trauma reported.
- Frequency of headaches: One point if not “nearly daily”.

### Descriptive statistics

The distribution of the candidate variables is visualized by histograms. The association between the variables is described by the Pearson correlation coefficient.

### Statistical methods for the confirmative part

The modifying effect of a candidate variable will be illustrated by reporting the mean values of the outcomes in each treatment group within each subpopulation defined by the candidate variable directly or after a suitable categorization. Categorizations will aim at defining three to five groups of equal size.

The estimated treatment effect at two anchor points will be visualized in a forest plot with 95% confidence intervals, and the modifying effect will be assessed as the difference between the effect at these two anchor points (interaction) with a 95% confidence interval and *p* value. The anchor points are chosen as the lower and upper 5th percentile of the candidate variable. The estimated treatment difference will be based on regression models including both the intervention variable and the candidate variables as well as the interaction term between the two. The baseline level is added as covariate where change scores are used as outcome (days with headache). The p-value of the interaction will be assessed with significance levels of both 5% and 10% to take the limited power into account.

### Statistical methods for the explorative part

We will use the technique of Tian et al. [35] to construct a new parsimonious index with modifying effect based on the full set of candidate variables given. The method is based on the simple idea to define a variable corresponding to the observed outcome in children exposed to chiropractic manipulation and to the negative of the observed outcome in children exposed to sham manipulation (after subtracting the overall mean value). Then the new index is based on trying to predict this variable based on the set of variables given. Twice the predicted value can be interpreted as the expected gain in using chiropractic manipulation instead of sham manipulation, and hence we will express the new index in these values. For constructing the new index, we will use ordinary regression combined with the lasso technique [36] as already suggested by Tian et al. [35]. This implies a variable selection, i.e., the lasso aims at constructing a parsimonious index with high modifying effect by selecting an optimal penalty parameter $\lambda$ penalizing the number of variables included. The optimal value is determined by cross validation. It should be noted that such an approach implies that among several correlated items, typically only one or a few are selected, and that this decision can be rather arbitrary. Hence it is essential to regard selected items as representatives of *constructs* which may modify the treatment effect.

The constructed new index assigns weights to the selected variables. In order to facilitate the interpretation of these weights, we use two different representations. The first refers to the weights when all variables have been standardized to a standard deviation of 1.0. This allows to compare weights across variables: the higher the weight, the stronger is the association of the new index with this variable. The second refers to the weight when the variables are not standardized. These can be interpreted directly as regression coefficients: increasing the variable by one point implies a change in the benefit from chiropractic manipulation by this coefficient.

In applying this approach, we neglected the candidate variable *Socioeconomic status*, as this was not available for many children, and the *Expected benefit index,* as this was already based on some of the original candidate variables.

## Results

### Distribution of candidate variables and association among candidate variables

**Fig. 1:**
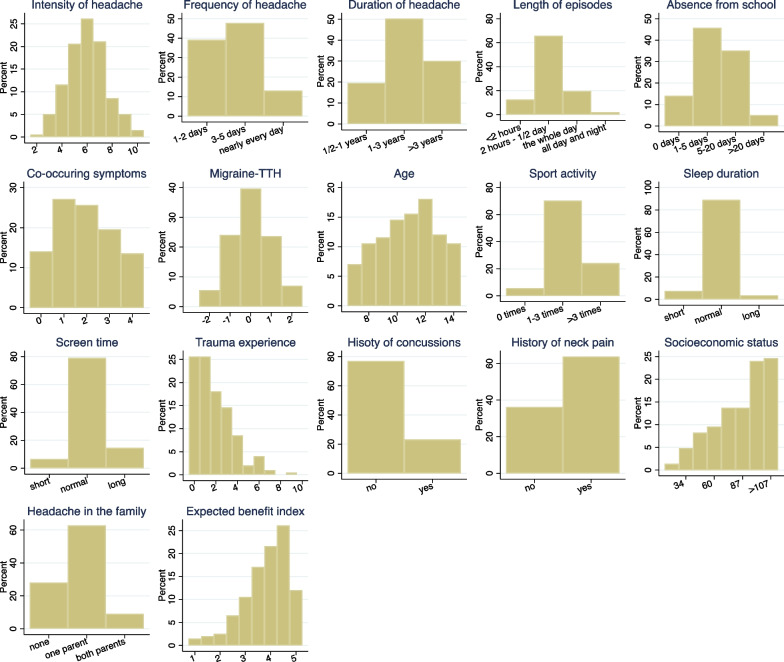
Distribution of the 17 candidate variables

Figure 1 shows the distribution of the 16 original candidate variables and the *expected benefit index*. Most variables showed a reasonable spread, except sleep duration and screen time, where the vast majority of children are in the middle group. However, some of the variables have a rather skewed distribution. The expected benefit index clearly distinguishes a few children with expectedly very unfavourable conditions from the majority of children with values around 3.5 to 4.5 and few children with a value of 5, i.e., satisfying all five conditions.

The associations among the candidate variables are shown in Additional file 3: Fig. S1. By construction, the *Expected benefit index* correlates with each of the five candidate variables defining the index. Similar, the *Migraine-tension-type index* correlates with several headache characteristics, in particular with *Co-occuring symptoms*. However, the other candidate variables are rather independent from each other, except for *Trauma experience* and *History of Concussions*. *Screen time* and *sleep duration* show the expected, negative correlation: Children with long screen times tend to have a low sleep duration.

### Confirmative part

**Fig. 2:**
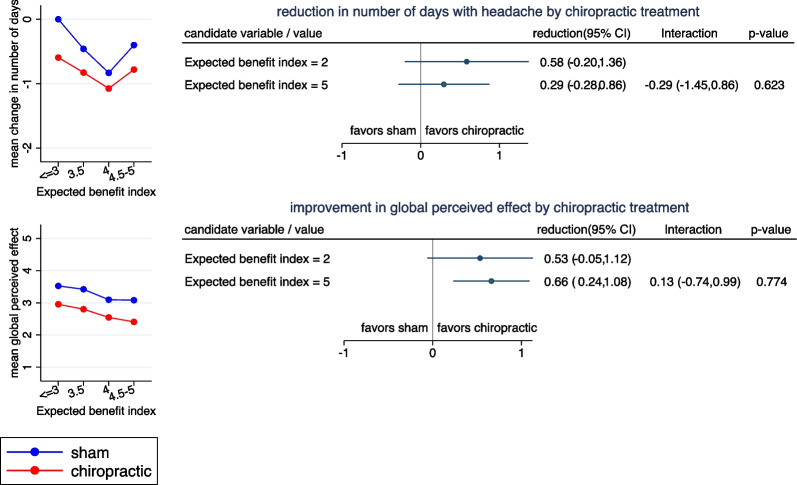
Reduction in number of days and GPE stratified by expected benefit index. Left side: The mean change in number of days with headache (upper panel) and the mean GPE (lower panel) stratified by treatment group and four subgroups of patients defined by the *Expected benefit index.* Right side: Estimated treatment effects (reduction in mean number of days with headache by chiropractic treatment (upper panel) and reduction in GPE) at two selected values of the *Expected benefit index*. The estimates are based on a model assuming a linear change of the treatment effect in dependence on the values. Interactions refer to the difference in reduction between the two selected values. Positive interactions indicate a more distinct advantage of chiropractic manipulation in case of the second (larger) value selected

Figure 2 depicts the association of the *Expected benefit index* with the treatment effect. After stratifying the children according to the index into four groups, within each group the outcomes are always lower (i.e., more favorable) under chiropractic treatment than under sham treatment, and there is little variation in the difference in mean values between the treatment groups (Left side of Fig. 2). Consequently, there are no distinct differences in the treatment effect (reduction in mean number of days with headache or in mean GPE) if children with a low index value are compared to children with a high index value (Right side of Fig. 2). There is little evidence for a modification of the treatment effect by the *Expected benefit index.*

**Fig. 3:**
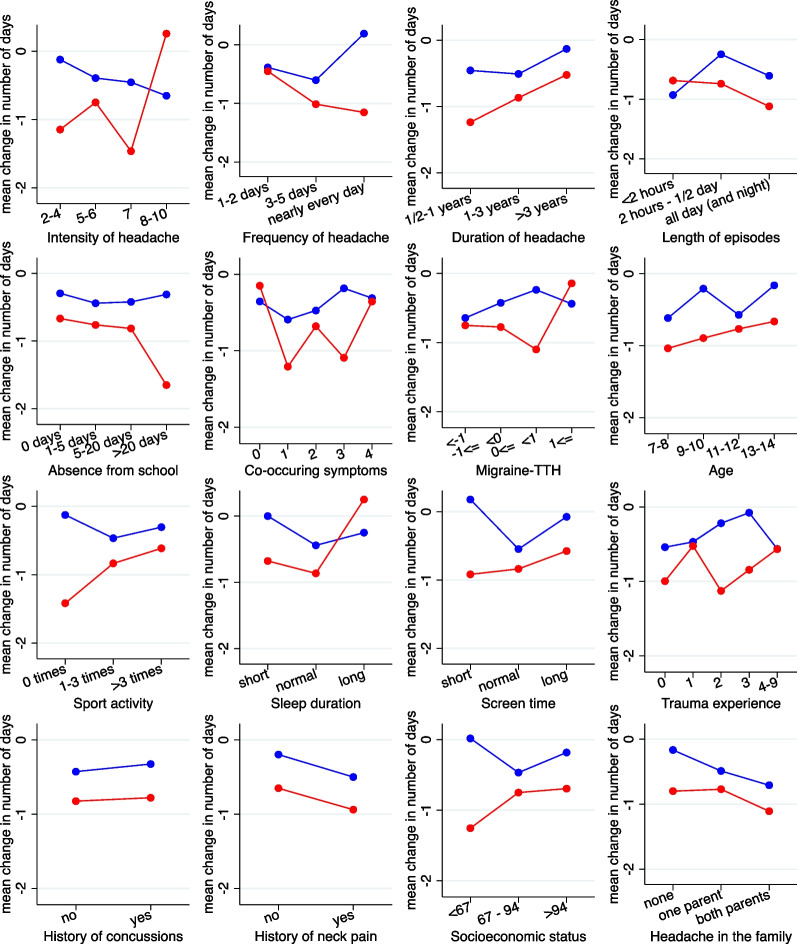
Change in number of headache-days stratified by treatment group for the 16 candidate variables. The candidate variables have been partially categorized to obtain three to five groups of roughly equal size. (red = chiropractic manipulation group; blue = sham manipulation group). TTH: Tension-type headache

**Fig. 4:**
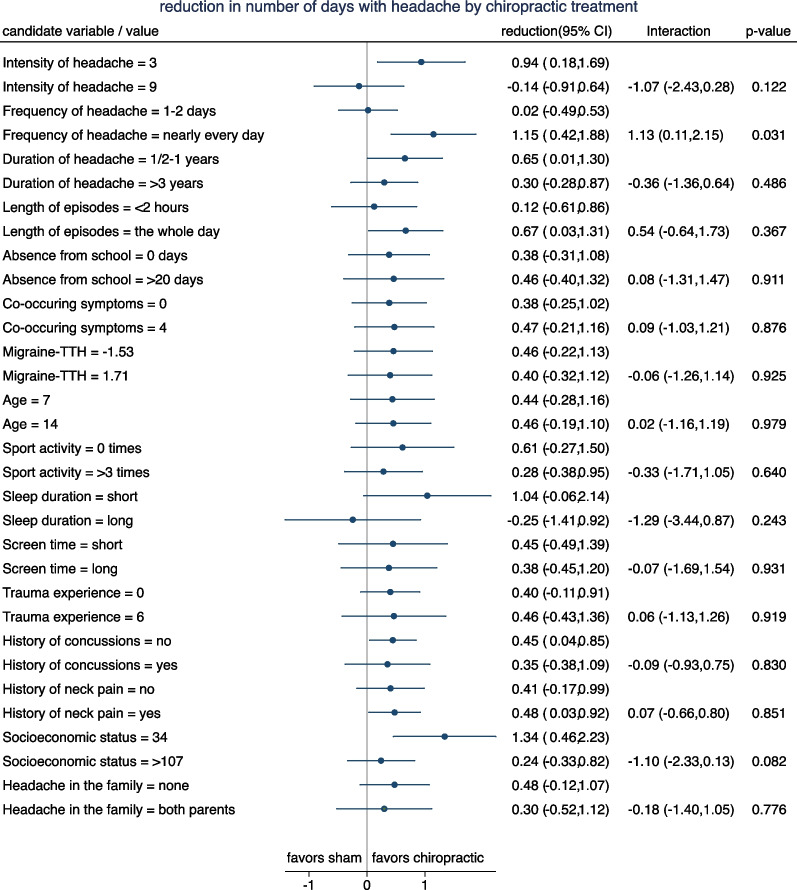
Reduction in mean number of headache-days at two selected values for each of candidate variables. The estimates are based on a model assuming a linear change of the treatment effect in dependence on the values. Interactions refer to the difference in reduction between the two selected values. Positive interactions indicate a more distinct advantage of chiropractic manipulation in case of the second (larger) value selected. TTH: Tension-type headache

Figures 3 and 4 depict the results for the original candidate variables with respect to number of days with headache. In Fig. 3 we can observe that the mean change in number of days is nearly always lower (i.e. more favorable) for chiropractic manipulation (red line) than for sham manipulation (blue line). There are only few candidate variables with a systematic trend in the difference between the two curves with increasing values of the candidate variables. According to Fig. 4 there are four candidate variables with a treatment effect differing by more than one day between the lower and the upper end of the spectrum of the candidate variable values*: Intensity of headache*, *Frequency of headache*, S*leep duration*, and *Socioeconomic status*. The interaction with *Frequency of headache* reached significance at the 5% level, and the interaction with *Socioeconomic status* at the 10% level.

**Fig. 5:**
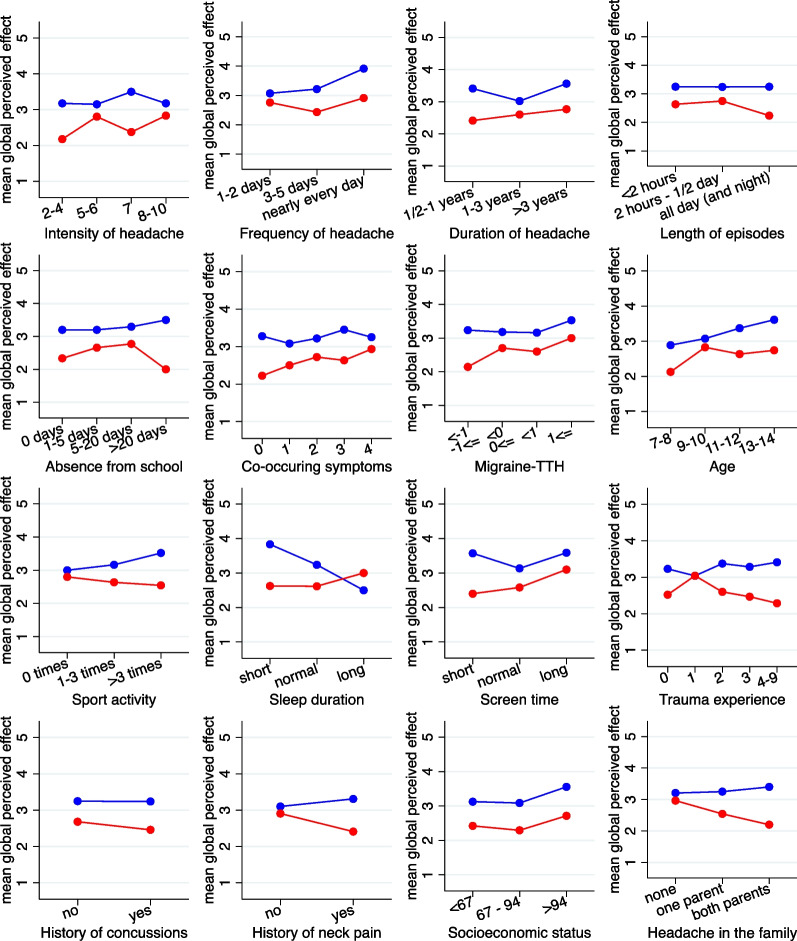
The mean GPE stratified by treatment group and 16 candidate variables. The candidate variables have been partially categorized to obtain three to five groups of roughly equal size. (red = chiropractic manipulation group; blue = sham manipulation group. GPE is coded with 1 = ’completely recovered’ and 7 = ’a lot worse’

**Fig. 6:**
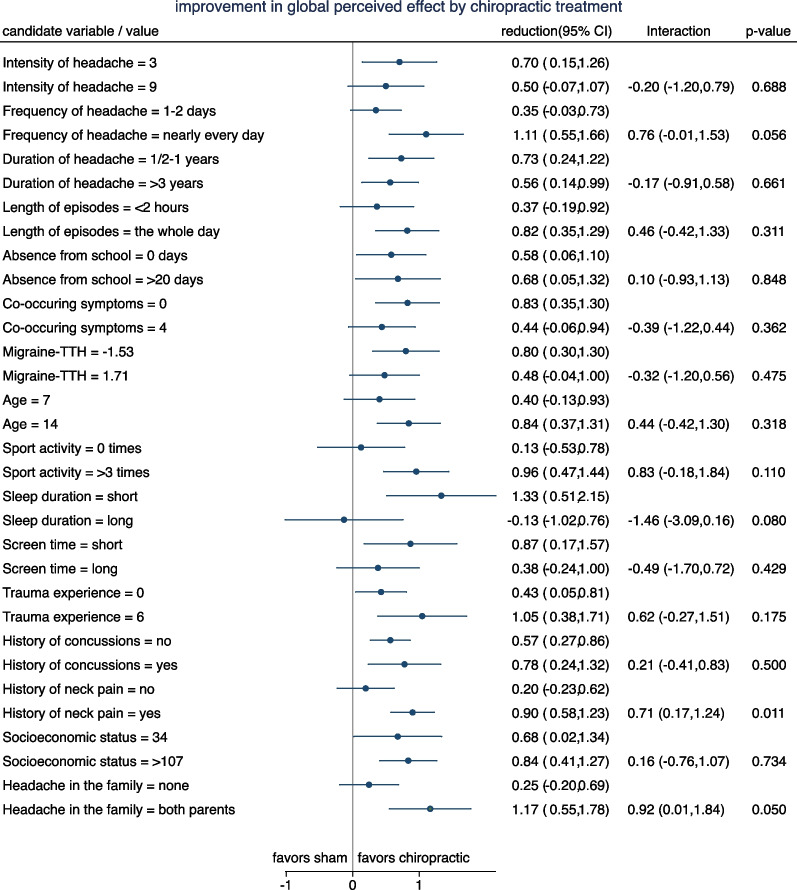
Reduction in mean GPE by chiropractic treatment at two selected values for each candidate variables. The estimates are based on a model assuming a linear change of the treatment effect in dependence on the values. Interactions refer to the difference in reduction between the two selected values. Positive interactions indicate a more distinct advantage of chiropractic manipulation in case of the second (larger) value selected

Figures 5 and 6 depict the results for the global perceived effect. In Fig. 5 we can observe that the GPE is nearly always lower (i.e. more favorable) for chiropractic manipulation (red line) than for sham manipulation (blue line). There are only few candidate variables with a systematic trend in the difference between the two curves with increasing values of the candidate variables. According to Fig. 6 there are five candidate variables with a treatment effect differing by more than 0.7 points between the lower and the upper end of the spectrum of the candidate variable values*: Frequency of headac*he, *Sport activity*, *Sleep duration*, *History of neck pain*, and *Headache in the family*. The interaction with *History of neck pain* and *Headache in the family* reached significance at the 5% level, and the interaction with *Frequency of headac*he and *Sleep duration* at the 10% level.

### Exploratory part

The attempt to construct a new index using the outcome *Change in number of days with headache* was not successful, i.e., the lasso selected no variables. Therefore, we only report the results with respect to *Global perceived effect*.

**Fig. 7:**
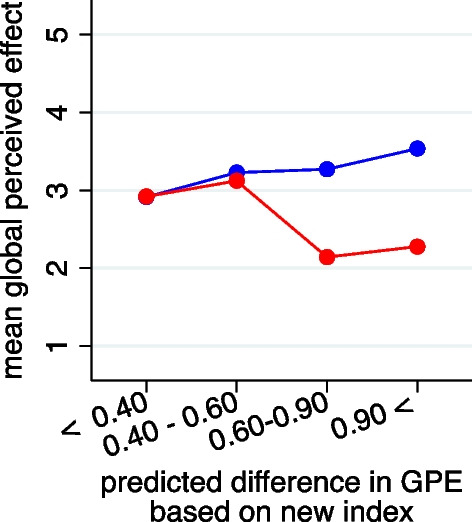
Mean GPE-change stratified by treatment group and the new index categorized into four groups. The values of the index refer to the predicted difference in GPE comparing chiropractic manipulation with sham manipulation. (red = chiropractic care; blue = control group)

When combining the 15 candidate variables into one parsimonious index, seven variables were selected based on the variable selection in the lasso technique, with weights shown in Table 2. The variables *History of neck pain* and *Headache in the family* got the highest weights. When grouping the values of the index, we can observe on the left side of Fig. 7 no treatment effect for children with low values of the index and a reduction of about 1 point on the GPE scale by chiropractic treatment for children with high values of the index.

**Table 2:** The weights given to the candidate variables in a new parsimonious index

Candidate variable	Weight	Effect
Frequency of headache	0.17	0.26
Length of episode	0.02	0.03
Sport activity	0.13	0.25
Sleep duration	− 0.13	− 0.40
Trauma experience	0.05	0.03
History of neck pain	0.21	0.43
Headache in the family	0.19	0.33

“Weight” refers to the regression coefficients when using the standardized variables as input. “Effect” refers to the regression coefficients when using the unstandardized variables as input

## Discussion

The results of the confirmative part of our investigation do not indicate that any of the candidate variables by themselves modify the effect of chiropractic treatment to a relevant degree. The observed treatment effects at selected anchor points ranged roughly from 0 to twice the observed overall effect, and probably mainly reflect random variation. This can be interpreted as a sign that chiropractic manipulation is offering an advantage for a broad range of children.

The results of the exploratory part suggest that there may be still a potential to identify subgroups of children with little benefit from chiropractic treatment or with a more pronounced benefit, respectively. However, we must have in mind that the differences observed are probably too optimistic as the same data set was used to construct the index and to evaluate it. Hence, the clinical relevance of this finding remains unclear unless future studies can corroborate these findings. Nevertheless, it might still be of interest to take a closer look at the variables which might be predictive of the benefit from chiropractic treatment.

The new index constructed included seven of the candidate variables. Three of them were already used by the principal investigator when representing her expectations of the highest and the lowest chance of a favorable outcome under chiropractic treatment: Frequency of headache, sport activity, and trauma experience. This can be seen as in concordance with the PIs experience. However, whereas we a priori regarded daily headache as an unfavorable condition for a benefit from chiropractic treatment, our analyses suggest an increasing benefit with increasing frequency. Such discrepancies with respect to the expected direction of the effect modification of single variables also explain the failure of the predefined expected benefit index to predict treatment effects. When correlating the five variables included in the index with the treatment effect on GPE, only trauma experience, screen time, and sport activity showed an association in the expected direction, whereas frequency of headache and age showed associations in the opposite direction.

If there are any subgroups of children benefitting from chiropractic treatment to a higher degree than other children, our results suggest that besides headache characteristics such as frequency and intensity, social factors may play a role: short sleep duration, low socio-economic status, and headache within the family may predict a higher benefit from chiropractic treatment. However, these relationships were not consistently observed over the two outcomes. This should be investigated further in future research considering that more than 70% of children with recurrent headaches have a family member with headache, predominantly the mother [19, 25, 34, 37] and children with family members with headache may be at risk of developing overuse of headache medication [34, 38]. Furthermore, in many Western countries, the reimbursement is considerably higher for pharmacological treatment than for non-pharmacological treatment, which means that children from homes with low income may choose pharmacological treatment due to the cost alone. This potential inequality of pediatric headache treatment should be addressed in future research.

Sport activity and a history of neck pain may be predictive for a higher benefit from chiropractic treatment, but this was only observed for the outcome of GPE. Trauma was also included in the new index resulting from the exploratory analysis, although only demonstrating minor modifying effect by itself. The measurement of trauma in the present study was rather superficial and future attention should be increased because headache symptoms after minor head and neck injuries may be delayed for months or years after the injury [26, 31, 39]. Thus, if previous trauma experience may be predictive for a higher benefit from chiropractic treatment, this could be an indicator for a chiropractic examination of children shortly after trauma experiences.

A basic limitation of our investigation is the sample size of the RCT. The sample size was chosen to establish an overall intervention effect. This implies a limited power to detect intervention effect modifiers. Furthermore, there have been no prior studies investigating effect modification in children with headache receiving chiropractic manipulation or sham manipulation. Consequently, we were forced to consider a rather broad spectrum of potential factors identified previously as important characteristic of different headache types or as potential risk factors. In addition, we rely on self-reports, which may only partially reflect the intended labeling. For example, according to the experience of the PI, some children might not report neck (or back) problems, although present, possibly because they have had it for so long that they consider it to be “normal”, or because the problems do not cause pain at the present time. In interpreting any observed effect modification, it must also be taken into account that the treatment provider was not blinded for the candidate variables. Thus, an estimated difference could be due to a more or less successful adaptation of the treatment to patient characteristics with respect to duration and intensity of the treatment.

Finally, it should be noted that the PI often expressed the expectation that chiropractic treatment shows a *faster* response in children with specific characteristics, which is not necessarily the same as having a better outcome at the end. This aspect will be examined in another paper.

## Conclusion

According to our current state of knowledge, chiropractic manipulation offers a moderate benefit for a broad spectrum of children. However, it cannot be excluded that specific headache characteristics, social factors, sport activity, or a history of neck pain may allow to identify children with an increased or a limited benefit. This question must be addressed in future studies.

## Supplementary Information

**Additional file 1. **Translated Baseline Questionnaire.

**Additional file 2. **Variable selection.

**Additional file 3. **Supplementary Figure 1. The pairwise correlations among the 17 candidate variables.

## Data Availability

Relevant anonymised data are available from the corresponding author on reasonable request.

## Abbreviations

GPE: Global perceived effect
NRS: Numerical rating scale
PI: Primary investigator
RCT: Randomized controlled trial
SMS: Short message service
TTH: Tension-type headache
