# Supplementary material for: Potential effect modifiers for treatment with chiropractic manipulation versus sham manipulation for recurrent headaches in children aged 7–14 years: development of and results from a secondary analysis of a randomised clinical trial
Source: Chiropr Man Therap. 2023 Jul 11;31:20. doi: 10.1186/s12998-023-00492-2 (PMC10337090; doi:10.1186/s12998-023-00492-2)
Supplement: Supplementary file 2 — Additional file 2. Variable selection. [file 12998_2023_492_MOESM2_ESM.pdf]

```
--------------------------------------------------------------------------------------------
      name:  <unnamed>
       log:  /Users/basler-akademie/work/nikkb/hovedpine/finaldata/hpmodify/data/prepare.l
> og
  log type:  text
 opened on:   2 Feb 2023, 17:20:03

. 
. *********************************************************************
. *** Process of constructing the candidate variables ****************
. *** The final candidate variables are starting with the letter I ***
. *** In addition, we collect the items to be considered in the ******
. *** exploratory part ***********************************************
. *********************************************************************
. *** Note: These analyses are based on all 279 children who filled **
. *** out the baseline questionnaire. Some of these children were  ***
. *** later not regarded as elegible for the RCT                   ***
. *********************************************************************
. 
. *********************************************************************
. *** Age *************************************************************
. *********************************************************************
. 
. *** Age in years is directly available as a variable
. 
. tab age

      Alder |      Freq.     Percent        Cum.
------------+-----------------------------------
          7 |         23        8.24        8.24
          8 |         30       10.75       19.00
          9 |         31       11.11       30.11
         10 |         40       14.34       44.44
         11 |         45       16.13       60.57
         12 |         42       15.05       75.63
         13 |         38       13.62       89.25
         14 |         30       10.75      100.00
------------+-----------------------------------
      Total |        279      100.00

. 
```

```
. gen Iage=age

. 
. local items  age

. 
. *******************************************************************
. **** Headache severity ********************************************
. *******************************************************************
. 
. ** There are four variables directly related to headache severity
. 
. tab1 frequency intensity duration length

-> tabulation of frequency_bl

   Hvor tit har du |
        hovedpine? |      Freq.     Percent        Cum.
-------------------+-----------------------------------
1 - 2 dage om ugen |        125       44.80       44.80
3 - 5 dage om ugen |        116       41.58       86.38
   Næsten hver dag |         38       13.62      100.00
-------------------+-----------------------------------
             Total |        279      100.00

-> tabulation of intensity

   Vælg det |
   tal, der |
      bedst |
  beskriver |
  graden af |
   din mest |
almindelige |
     smerte |      Freq.     Percent        Cum.
------------+-----------------------------------
          2 |          2        0.72        0.72
          3 |         18        6.45        7.17
          4 |         31       11.11       18.28
          5 |         57       20.43       38.71
          6 |         67       24.01       62.72
          7 |         59       21.15       83.87
          8 |         28       10.04       93.91
          9 |         10        3.58       97.49
         10 |          7        2.51      100.00
------------+-----------------------------------
      Total |        279      100.00

-> tabulation of duration_bl
```

Hvor længe har du haft hovedpine?	Freq.	Percent	Cum.
½ - 1 år	64	22.94	22.94
1 - 3 år	137	49.10	72.04
Mere end 3 år	78	27.96	100.00
Total	279	100.00	

```
-> tabulation of length
```

Hvor lang tid varer din hovedpine typisk?	Freq.	Percent	Cum.
Mindre end 2 timer	32	11.47	11.47
Fra 2 timer op til ½ dag	189	67.74	79.21
Hele dagen	52	18.64	97.85
Hele dagen og natten	6	2.15	100.00
Total	279	100.00	

```
.
. ** In addition, we can use the use of headache medicine:
.
. tab1 nonpremed premedicine_bl premedicinefreq_bl

-> tabulation of nonpremedicine_bl
```

Hvor ofte tager du håndkøbsmedicin for hovedpine (fx panodil eller ipren)?	Freq.	Percent	Cum.
Aldrig	32	11.47	11.47
1 - 3 gange om måneden	157	56.27	67.74
1 - 3 gange om ugen	80	28.67	96.42
Mere end 3 gange om ugen	10	3.58	100.00
Total	279	100.00	

```
-> tabulation of premedicine_bl
```

Tager du receptpligtig medcicin for din hovedpine?	Freq.	Percent	Cum.
Nej	267	95.70	95.70
Ja	12	4.30	100.00
Total	279	100.00	

-> tabulation of premedicinefreq_bl

Hvor ofte tager du det?	Freq.	Percent	Cum.
1 - 3 gange om måneden	8	66.67	66.67
1 - 3 gange om ugen	1	8.33	75.00
Mere end 3 gange om ugen	3	25.00	100.00
Total	12	100.00	

.
. tab nonpremed premedicine_bl

Hvor ofte tager du håndkøbsmedicin for hovedpine (fx panodil eller ipren)?	Tager du receptpligtig medcicin for din hovedpine? Nej	Ja	Total
Aldrig	32	0	32
1 - 3 gange om månede	152	5	157
1 - 3 gange om ugen	74	6	80
Mere end 3 gange om u	9	1	10
Total	267	12	279

. tab nonpremed premedicinefreq_bl

Hvor ofte tager du håndkøbsmedicin for hovedpine (fx panodil eller ipren)?	Hvor ofte tager du det? 1 - 3 gan	1 - 3 gan	Mere end	Total
1 - 3 gange om månede	4	0	1	5
1 - 3 gange om ugen	4	1	1	6
Mere end 3 gange om u	0	0	1	1
Total	8	1	3	12

```
.
. ** It is not straightforward how to combine these two variables into one.
. ** However, as there are only few children with prescribed medicine,
. ** we combine this into one variable by taking the maximum entry of both
.
. egen medication = rowmax(nonpremed premedicinefreq_bl)

.
. mytablist nonpremed premedicine_bl premedicinefreq_bl medication

     +---------------------------------------------------------------------------------+
     |         nonpremedicine_bl   pre~e_bl          premedicinefreq_bl   medica~n   freq |
     |---------------------------------------------------------------------------------|
  1. |                    Aldrig        Nej                           .          1     32 |
  2. |   1 - 3 gange om måneden        Nej                           .          2    152 |
  3. |   1 - 3 gange om måneden         Ja      1 - 3 gange om måneden          2      4 |
  4. |   1 - 3 gange om måneden         Ja   Mere end 3 gange om ugen          4      1 |
  5. |      1 - 3 gange om ugen        Nej                           .          3     74 |
     |---------------------------------------------------------------------------------|
  6. |      1 - 3 gange om ugen         Ja      1 - 3 gange om måneden          3      4 |
  7. |      1 - 3 gange om ugen         Ja         1 - 3 gange om ugen          3      1 |
  8. |      1 - 3 gange om ugen         Ja   Mere end 3 gange om ugen          4      1 |
  9. | Mere end 3 gange om ugen        Nej                           .          4      9 |
 10. | Mere end 3 gange om ugen         Ja   Mere end 3 gange om ugen          4      1 |
     +---------------------------------------------------------------------------------+

.
.
. ** A further variable which might be informative here is the number of
. ** sick days because of headache
.
. tab sickdaysHA

  Hvor mange sygedage |
     har du haft pga. |
hovedpine det seneste |
                  år? |      Freq.     Percent        Cum.
----------------------+-----------------------------------
           0 sygedage |         44       15.77       15.77
       1 - 5 sygedage |        120       43.01       58.78
      5 - 20 sygedage |         97       34.77       93.55
Flere end 20 sygedage |         18        6.45      100.00
----------------------+-----------------------------------
                Total |        279      100.00
```

```
.
. *** Note however, that these variables are only moderately associated
. *** with each other:
.
. pwcorr frequency intensity duration length medication sickdaysHA

             | freque~l intens~y durati~l   length medica~n sickda~A
-------------+------------------------------------------------------
frequency_bl |   1.0000
   intensity |  -0.0925   1.0000
 duration_bl |   0.0026   0.1643   1.0000
      length |   0.1090   0.1322   0.0774   1.0000
  medication |   0.0155   0.1326   0.1519   0.0730   1.0000
  sickdaysHA |   0.0616   0.2545   0.1891   0.2291   0.1696   1.0000


.
. local items `items' frequency intensity duration length nonpremed premedicinefreq_bl sic
> kdaysHA


.
. ** Interestingly, sickdays seem to correlate somewhat with everything,
. ** and frequency does not seem to correlate with the other variables
. ** (except of length).
.
. ** Note: The correlation between frequency and intensity is actually
. ** negative, but this is not statistically significant:
.
. pwcorr frequency intensity, sig

             | freque~l intens~y
-------------+------------------
frequency_bl |   1.0000
             |
             |
   intensity |  -0.0925   1.0000
             |   0.1231
             |


.
.
. ** This suggests already to reduce the severity variables to two factors.
```

```
. ** This is corroborated by a factor analysis with varimax rotation:
.
. qui factor frequency intensity duration  medication length sickdaysHA

. rotate

Factor analysis/correlation                        Number of obs    =        279
    Method: principal factors                      Retained factors =          3
    Rotation: orthogonal varimax (Kaiser off)      Number of params =         15

    --------------------------------------------------------------------------
         Factor  |     Variance   Difference        Proportion   Cumulative
    -------------+------------------------------------------------------------
        Factor1  |      0.73094      0.56635            1.6157       1.6157
        Factor2  |      0.16459      0.16281            0.3638       1.9795
        Factor3  |      0.00179            .            0.0040       1.9834
    --------------------------------------------------------------------------
    LR test: independent vs. saturated:  chi2(15) =   71.56 Prob>chi2 = 0.0000

Rotated factor loadings (pattern matrix) and unique variances

    -----------------------------------------------------------
        Variable |  Factor1   Factor2   Factor3 |   Uniqueness 
    -------------+------------------------------+--------------
    frequency_bl |   0.0043    0.2965    0.0042 |      0.9120  
       intensity |   0.4291   -0.0886   -0.0021 |      0.8080  
     duration_bl |   0.3510   -0.0214    0.0261 |      0.8756  
      medication |   0.3139    0.0037    0.0300 |      0.9005  
          length |   0.3006    0.2302   -0.0135 |      0.8564  
      sickdaysHA |   0.4844    0.1240    0.0027 |      0.7500  
    -----------------------------------------------------------

Factor rotation matrix

    -----------------------------------------
                 | Factor1  Factor2  Factor3 
    -------------+---------------------------
         Factor1 |  0.9889   0.1471   0.0195 
         Factor2 | -0.1465   0.9889  -0.0264 
         Factor3 | -0.0232   0.0232   0.9995 
    -----------------------------------------
```

```
.
.
. ** This suggest one factor related to all variables except of frequency,
. ** and one factor related mainly to frequency and length.
. ** (This is also in line with the migraine-tension distinction, where intensity
. ** plays a major role, wheresa the role of frequency is less clear)
.
. ** In order to arrive at two conceptually non-overlapping variables,
. ** we decided to combine frequency and length into an index reflecting the
. ** time spent with headache and the remaining four variables into an index
. ** reflecting severity.
.
. ** For the latter, we use the first PCA score:
.
. pca intensity duration  medication sickdaysHA

Principal components/correlation                 Number of obs    =        279
                                                 Number of comp.  =          4
                                                 Trace            =          4
    Rotation: (unrotated = principal)            Rho              =     1.0000

    --------------------------------------------------------------------------
       Component |   Eigenvalue   Difference         Proportion   Cumulative
    -------------+------------------------------------------------------------
           Comp1 |      1.53598      .651847             0.3840       0.3840
           Comp2 |      .884131     .0449141             0.2210       0.6050
           Comp3 |      .839217     .0985431             0.2098       0.8148
           Comp4 |      .740674            .             0.1852       1.0000
    --------------------------------------------------------------------------

Principal components (eigenvectors)

    --------------------------------------------------------------------
        Variable |    Comp1     Comp2     Comp3     Comp4 | Unexplained
    -------------+----------------------------------------+-------------
       intensity |   0.5190   -0.5230   -0.2441    0.6305 |           0
     duration_bl |   0.4793    0.1726    0.8567    0.0803 |           0
      medication |   0.4397    0.7855   -0.4162    0.1284 |           0
      sickdaysHA |   0.5546   -0.2825   -0.1820   -0.7613 |           0
    --------------------------------------------------------------------
```

```
. predict aux
(score assumed)
(3 components skipped)

Scoring coefficients
    sum of squares(column-loading) = 1

    ------------------------------------------------------
        Variable |    Comp1     Comp2     Comp3     Comp4 
    -------------+----------------------------------------
       intensity |   0.5190   -0.5230   -0.2441    0.6305 
     duration_bl |   0.4793    0.1726    0.8567    0.0803 
      medication |   0.4397    0.7855   -0.4162    0.1284 
      sickdaysHA |   0.5546   -0.2825   -0.1820   -0.7613 
    ------------------------------------------------------

. egen HAseverity = std(aux)

. drop aux

. 
. 
. *** As expected, this score is not related to frequency
. 
. pwcorr HAseverity frequency length

             | HAseve~y freque~l   length
-------------+---------------------------
  HAseverity |   1.0000 
frequency_bl |  -0.0047   1.0000 
      length |   0.2137   0.1090   1.0000 

. 
. ** Combining frequency and length makes some sense, as both variables together
. ** define the "time with headache per week". Logically, this would suggest to
. ** multiply these two numbers, but as frequency and length are based on categories,
. ** it is less obvious whether the product makes sense. We take here a look at both:
. 
. 
. gen hatime1 = frequency + length -2
```

```
. gen hatime2 = frequency * length -1

.
. tab1 hatime*

-> tabulation of hatime1

    hatime1 |      Freq.     Percent        Cum.
------------+-----------------------------------
          0 |         17        6.09        6.09
          1 |         98       35.13       41.22
          2 |        102       36.56       77.78
          3 |         49       17.56       95.34
          4 |         11        3.94       99.28
          5 |          2        0.72      100.00
------------+-----------------------------------
      Total |        279      100.00

-> tabulation of hatime2

    hatime2 |      Freq.     Percent        Cum.
------------+-----------------------------------
          0 |         17        6.09        6.09
          1 |         98       35.13       41.22
          2 |         24        8.60       49.82
          3 |         79       28.32       78.14
          5 |         48       17.20       95.34
          7 |          3        1.08       96.42
          8 |          8        2.87       99.28
         11 |          2        0.72      100.00
------------+-----------------------------------
      Total |        279      100.00

.
. pwcorr  hatime*

             |  hatime1  hatime2
-------------+------------------
     hatime1 |   1.0000
     hatime2 |   0.9719   1.0000
```

```
.
. ** The variables are highly correlated. As hatime2 has a somewhat skewed
. ** distribution,  and the argument to multiply the two variables is not very
. ** convincing, we decided to use the first definition to define a
. ** headache time index
.
. rename hatime1 HAtime

. drop hatime2


.
. ** Note that the two indices are nearly uncorrelated.
.
. pwcorr HA*

             | HAseve~y   HAtime
-------------+------------------
  HAseverity |   1.0000 
      HAtime |   0.1304   1.0000 


.
.
. ***********************************************************************
. *** migraine-tension-type index ***************************************
. ***********************************************************************
.
. ** Dissing et al considered besides the severity index defined above also
. ** the following four single symptoms relevant for the migraine/tension-type
. ** distinction:
.
. tab1 symp_nausea symp_vomit symp_lightsens symp_soundsens

-> tabulation of symp_nausea_bl  

     Har du |
      andre |
  symptomer |
 sammen med |
       dine |
hovedpinean |
    fald? - |
     Kvalme |      Freq.     Percent        Cum.
------------+-----------------------------------
        Nej |        133       47.67       47.67
         Ja |        146       52.33      100.00
------------+-----------------------------------
      Total |        279      100.00

-> tabulation of symp_vomit_bl  
```

```
     Har du |
      andre |
  symptomer |
 sammen med |
       dine |
hovedpinean |
    fald? - |
     Opkast |      Freq.     Percent        Cum.
------------+-----------------------------------
        Nej |        219       78.49       78.49
         Ja |         60       21.51      100.00
------------+-----------------------------------
      Total |        279      100.00

-> tabulation of symp_lightsensi_bl

     Har du |
      andre |
  symptomer |
 sammen med |
       dine |
hovedpinean |
    fald? - |
 Lysfølsomh |
         ed |      Freq.     Percent        Cum.
------------+-----------------------------------
        Nej |        125       44.80       44.80
         Ja |        154       55.20      100.00
------------+-----------------------------------
      Total |        279      100.00

-> tabulation of symp_soundsensi_bl

     Har du |
      andre |
  symptomer |
 sammen med |
       dine |
hovedpinean |
    fald? - |
 Lydfølsomh |
         ed |      Freq.     Percent        Cum.
------------+-----------------------------------
        Nej |        109       39.07       39.07
         Ja |        170       60.93      100.00
------------+-----------------------------------
      Total |        279      100.00
```

```
.
. local items `items' symp_nausea symp_vomit symp_lightsens symp_soundsens

.
. ** All four symptoms to be rather distinctly associated
.
. tab2 symp_nausea symp_vomit symp_lightsens symp_soundsens, row exact

-> tabulation of symp_nausea_bl by symp_vomit_bl

+----------------+
| Key            |
|----------------|
|   frequency    |
| row percentage |
+----------------+

    Har du |
     andre |
 symptomer |
sammen med |     Har du andre
      dine | symptomer sammen med
hovedpinea | dine hovedpineanfald?
  nfald? - |       - Opkast
    Kvalme |       Nej         Ja |     Total
-----------+----------------------+----------
       Nej |       131          2 |       133
           |     98.50       1.50 |    100.00
-----------+----------------------+----------
        Ja |        88         58 |       146
           |     60.27      39.73 |    100.00
-----------+----------------------+----------
     Total |       219         60 |       279
           |     78.49      21.51 |    100.00

           Fisher's exact =                 0.000
   1-sided Fisher's exact =                 0.000

-> tabulation of symp_nausea_bl by symp_lightsensi_bl

+----------------+
| Key            |
|----------------|
|   frequency    |
| row percentage |
+----------------+
```

```
    Har du |
     andre |
 symptomer |
sammen med |     Har du andre
      dine | symptomer sammen med
hovedpinea | dine hovedpineanfald?
  nfald? - |    - Lysfølsomhed
    Kvalme |       Nej         Ja |     Total
-----------+----------------------+----------
       Nej |        74         59 |       133
           |     55.64      44.36 |    100.00
-----------+----------------------+----------
        Ja |        51         95 |       146
           |     34.93      65.07 |    100.00
-----------+----------------------+----------
     Total |       125        154 |       279
           |     44.80      55.20 |    100.00

           Fisher's exact =                 0.001
   1-sided Fisher's exact =                 0.000

-> tabulation of symp_nausea_bl by symp_soundsensi_bl

+-----------------+
| Key             |
|-----------------|
|   frequency     |
| row percentage  |
+-----------------+

    Har du |
     andre |
 symptomer |
sammen med |     Har du andre
      dine | symptomer sammen med
hovedpinea | dine hovedpineanfald?
  nfald? - |    - Lydfølsomhed
    Kvalme |       Nej         Ja |     Total
-----------+----------------------+----------
       Nej |        66         67 |       133
           |     49.62      50.38 |    100.00
-----------+----------------------+----------
        Ja |        43        103 |       146
           |     29.45      70.55 |    100.00
-----------+----------------------+----------
     Total |       109        170 |       279
           |     39.07      60.93 |    100.00

           Fisher's exact =                 0.001
   1-sided Fisher's exact =                 0.000

-> tabulation of symp_vomit_bl by symp_lightsensi_bl
```

```
+-----------------+
| Key             |
|-----------------|
|   frequency     |
| row percentage  |
+-----------------+

    Har du |
     andre |
 symptomer |
sammen med |     Har du andre
      dine | symptomer sammen med
hovedpinea | dine hovedpineanfald?
  nfald? - |    - Lysfølsomhed
    Opkast |       Nej         Ja |     Total
-----------+----------------------+----------
       Nej |       116        103 |       219
           |     52.97      47.03 |    100.00
-----------+----------------------+----------
        Ja |         9         51 |        60
           |     15.00      85.00 |    100.00
-----------+----------------------+----------
     Total |       125        154 |       279
           |     44.80      55.20 |    100.00

           Fisher's exact =                 0.000
   1-sided Fisher's exact =                 0.000

-> tabulation of symp_vomit_bl by symp_soundsensi_bl

+-----------------+
| Key             |
|-----------------|
|   frequency     |
| row percentage  |
+-----------------+

    Har du |
     andre |
 symptomer |
sammen med |     Har du andre
      dine | symptomer sammen med
hovedpinea | dine hovedpineanfald?
  nfald? - |    - Lydfølsomhed
    Opkast |       Nej         Ja |     Total
-----------+----------------------+----------
       Nej |        92        127 |       219
           |     42.01      57.99 |    100.00
-----------+----------------------+----------
        Ja |        17         43 |        60
           |     28.33      71.67 |    100.00
-----------+----------------------+----------
     Total |       109        170 |       279
```

```
           |     39.07      60.93 |    100.00

           Fisher's exact =                 0.073
   1-sided Fisher's exact =                 0.037

-> tabulation of symp_lightsensi_bl by symp_soundsensi_bl

+-----------------+
| Key             |
|-----------------|
|    frequency    |
| row percentage  |
+-----------------+

    Har du |
     andre |
 symptomer |
sammen med |
      dine |     Har du andre
hovedpinea | symptomer sammen med
  nfald? - | dine hovedpineanfald?
 Lysfølsom |    - Lydfølsomhed
       hed |       Nej         Ja |     Total
-----------+----------------------+----------
       Nej |        72         53 |       125
           |     57.60      42.40 |    100.00
-----------+----------------------+----------
        Ja |        37        117 |       154
           |     24.03      75.97 |    100.00
-----------+----------------------+----------
     Total |       109        170 |       279
           |     39.07      60.93 |    100.00

           Fisher's exact =                 0.000
   1-sided Fisher's exact =                 0.000

.
. foreach var1 of varlist symp_nausea symp_vomit symp_lightsens symp_soundsens {
  2.   if "`ferest()'"!="" {
  3.     foreach var2 of varlist `ferest()' {
  4.           qui cc `var1' `var2'
  5.           di "`var1' `var2' : " _col(44) r(or)
  6.         }
  7.   }
  8. }
symp_nausea_bl symp_vomit_bl :             43.170455
symp_nausea_bl symp_lightsensi_bl :        2.3363244
symp_nausea_bl symp_soundsensi_bl :        2.3595974
symp_vomit_bl symp_lightsensi_bl :         6.381877
symp_vomit_bl symp_soundsensi_bl :         1.8323298
symp_lightsensi_bl symp_soundsensi_bl :    4.2957675
```

```
.
. ** Consequently, Dissing et al defined a headache symptom index:
.
. egen IHAsymptoms  = rowtotal(symp_nausea symp_vomit symp_lightsens symp_soundsens) , mis
> sing

.
. tab IHAsymptoms

IHAsymptoms |      Freq.     Percent        Cum.
------------+-----------------------------------
          0 |         45       16.13       16.13
          1 |         71       25.45       41.58
          2 |         68       24.37       65.95
          3 |         57       20.43       86.38
          4 |         38       13.62      100.00
------------+-----------------------------------
      Total |        279      100.00

.
. *** As pointed out by Dissing et al, the severity and the symtom index are correlated.
.
. pwcorr IHAsymptoms HAseverity

             | IHAsym~s HAseve~y
-------------+------------------
 IHAsymptoms |   1.0000
  HAseverity |   0.4509   1.0000

.
. ** The migraine/tension distinction involves also the aspect of
. ** aggrevation/avoidance by physical activity. This can be reflectd by the
. ** variables "cause_sports" , "help_sports" , "help_lying" and "help_sleep".
. ** Indeed, there is a very clear association of the symptom index with
. ** the last two help_variables and also with cause_sports:
.
. tab IHAsymptoms cause_sports, row

+----------------+
| Key            |
|----------------|
|   frequency    |
| row percentage |
+----------------+
```

```
           |  Kan én eller flere
           |  af disse ting give
           |   dig hovedpine? -
IHAsymptom |         Sport
         s |       Nej         Ja |     Total
-----------+----------------------+----------
         0 |        37          8 |        45 
           |     82.22      17.78 |    100.00 
-----------+----------------------+----------
         1 |        57         14 |        71 
           |     80.28      19.72 |    100.00 
-----------+----------------------+----------
         2 |        47         21 |        68 
           |     69.12      30.88 |    100.00 
-----------+----------------------+----------
         3 |        38         19 |        57 
           |     66.67      33.33 |    100.00 
-----------+----------------------+----------
         4 |        27         11 |        38 
           |     71.05      28.95 |    100.00 
-----------+----------------------+----------
     Total |       206         73 |       279 
           |     73.84      26.16 |    100.00 

. 
. logit cause_sports IHAsymptoms

Iteration 0:   log likelihood = -160.36206  
Iteration 1:   log likelihood = -158.46646  
Iteration 2:   log likelihood = -158.45902  
Iteration 3:   log likelihood = -158.45902  

Logistic regression                                     Number of obs =    279
                                                        LR chi2(1)    =   3.81
                                                        Prob > chi2   = 0.0511
Log likelihood = -158.45902                             Pseudo R2     = 0.0119

--------------------------------------------------------------------------------
cause_sports_bl | Coefficient  Std. err.      z    P>|z|     [95% conf. interval]
----------------+---------------------------------------------------------------
    IHAsymptoms |   .2083947   .1074963     1.94   0.053    -.0022942    .4190836
          _cons |  -1.450105   .2604618    -5.57   0.000    -1.960601   -.9396093
--------------------------------------------------------------------------------
```

```
.
. tab IHAsymptoms help_sports , row

+----------------+
| Key            |
|----------------|
|   frequency    |
| row percentage |
+----------------+

           |  Kan én eller fleres
           | af disse ting hjælpe
           |  på din hovedpine? -
IHAsymptom |        Sport
         s |       Nej         Ja |     Total
-----------+----------------------+----------
         0 |        39          6 |        45 
           |     86.67      13.33 |    100.00 
-----------+----------------------+----------
         1 |        61         10 |        71 
           |     85.92      14.08 |    100.00 
-----------+----------------------+----------
         2 |        57         11 |        68 
           |     83.82      16.18 |    100.00 
-----------+----------------------+----------
         3 |        50          7 |        57 
           |     87.72      12.28 |    100.00 
-----------+----------------------+----------
         4 |        34          4 |        38 
           |     89.47      10.53 |    100.00 
-----------+----------------------+----------
     Total |       241         38 |       279 
           |     86.38      13.62 |    100.00 

.
. logit help_sports IHAsymptoms

Iteration 0:   log likelihood = -111.04375
Iteration 1:   log likelihood =  -110.9492
Iteration 2:   log likelihood = -110.94912
Iteration 3:   log likelihood = -110.94912

Logistic regression                               Number of obs =    279
                                                  LR chi2(1)    =   0.19
                                                  Prob > chi2   = 0.6635
Log likelihood = -110.94912                       Pseudo R2     = 0.0009
```

```
--------------------------------------------------------------------------------
help_sports_bl | Coefficient  Std. err.      z    P>|z|     [95% conf. interval]
---------------+----------------------------------------------------------------
   IHAsymptoms |  -.0594884   .1369916    -0.43   0.664    -.3279869    .2090102
         _cons |   -1.73631   .3053826    -5.69   0.000    -2.334849   -1.137772
--------------------------------------------------------------------------------

.
. tab IHAsymptoms help_lying , row

+----------------+
| Key            |
|----------------|
|   frequency    |
| row percentage |
+----------------+

           |  Kan én eller fleres
           | af disse ting hjælpe
           |  på din hovedpine? -
IHAsymptom |     At ligge ned
         s |       Nej         Ja |     Total
-----------+----------------------+----------
         0 |        28         17 |        45 
           |     62.22      37.78 |    100.00 
-----------+----------------------+----------
         1 |        35         36 |        71 
           |     49.30      50.70 |    100.00 
-----------+----------------------+----------
         2 |        30         38 |        68 
           |     44.12      55.88 |    100.00 
-----------+----------------------+----------
         3 |        19         38 |        57 
           |     33.33      66.67 |    100.00 
-----------+----------------------+----------
         4 |         9         29 |        38 
           |     23.68      76.32 |    100.00 
-----------+----------------------+----------
     Total |       121        158 |       279 
           |     43.37      56.63 |    100.00 
```

```
.
. logit help_lying IHAsymptoms

Iteration 0:   log likelihood = -190.92742
Iteration 1:   log likelihood = -182.97674
Iteration 2:   log likelihood = -182.95562
Iteration 3:   log likelihood = -182.95562

Logistic regression                                     Number of obs =    279
                                                        LR chi2(1)    =  15.94
                                                        Prob > chi2   = 0.0001
Log likelihood = -182.95562                             Pseudo R2     = 0.0418

----------------------------------------------------------------------------------
help_lyingdown_bl | Coefficient  Std. err.      z    P>|z|     [95% conf. interval]
------------------+---------------------------------------------------------------
      IHAsymptoms |   .3889321   .1004017     3.87   0.000     .1921484    .5857158
            _cons |  -.4548075   .2201962    -2.07   0.039     -.886384    -.023231
----------------------------------------------------------------------------------


.
. tab IHAsymptoms help_sleep , row

+----------------+
| Key            |
|----------------|
|   frequency    |
| row percentage |
+----------------+

           |  Kan én eller fleres
           | af disse ting hjælpe
           |  på din hovedpine? -
IHAsymptom |        At sove
         s |       Nej         Ja |     Total
-----------+----------------------+----------
         0 |        15         30 |        45
           |     33.33      66.67 |    100.00
-----------+----------------------+----------
         1 |        14         57 |        71
           |     19.72      80.28 |    100.00
-----------+----------------------+----------
         2 |        11         57 |        68
           |     16.18      83.82 |    100.00
-----------+----------------------+----------
         3 |        12         45 |        57
           |     21.05      78.95 |    100.00
-----------+----------------------+----------
         4 |         1         37 |        38
           |      2.63      97.37 |    100.00
-----------+----------------------+----------
     Total |        53        226 |       279
           |     19.00      81.00 |    100.00
```

```
.
. logit help_sleep IHAsymptoms

Iteration 0:   log likelihood = -135.64171
Iteration 1:   log likelihood = -131.27665
Iteration 2:   log likelihood = -131.18612
Iteration 3:   log likelihood = -131.18605
Iteration 4:   log likelihood = -131.18605

Logistic regression                                     Number of obs =    279
                                                        LR chi2(1)    =   8.91
                                                        Prob > chi2   = 0.0028
Log likelihood = -131.18605                             Pseudo R2     = 0.0328

----------------------------------------------------------------------------------
help_sleeping_bl | Coefficient  Std. err.      z    P>|z|     [95% conf. interval]
-----------------+----------------------------------------------------------------
     IHAsymptoms |   .3697539   .1279406     2.89   0.004      .118995    .6205128
           _cons |   .8159509   .2505384     3.26   0.001     .3249046    1.306997
----------------------------------------------------------------------------------

.
. ** There is, however,  no distinct associations between these three variables:
.
. tab2 cause_sports help_lying help_sleep , row

-> tabulation of cause_sports_bl by help_lyingdown_bl

+----------------+
| Key            |
|----------------|
|   frequency    |
| row percentage |
+----------------+

    Kan én |
     eller |
   flere af |  Kan én eller fleres
disse ting | af disse ting hjælpe
  give dig |  på din hovedpine? -
hovedpine? |     At ligge ned
   - Sport |       Nej         Ja |     Total
-----------+----------------------+----------
       Nej |        92        114 |       206
           |     44.66      55.34 |    100.00
-----------+----------------------+----------
        Ja |        29         44 |        73
           |     39.73      60.27 |    100.00
-----------+----------------------+----------
     Total |       121        158 |       279
           |     43.37      56.63 |    100.00
```

-> tabulation of cause_sports_bl by help_sleeping_bl

Key
frequency
row percentage

Kan én eller flere af disse ting give dig hovedpine? - Sport	Kan én eller flere af disse ting hjælpe på din hovedpine? - At sove		
	Nej	Ja	Total
Nej	37	169	206
	17.96	82.04	100.00
Ja	16	57	73
	21.92	78.08	100.00
Total	53	226	279
	19.00	81.00	100.00

-> tabulation of help_lyingdown_bl by help_sleeping_bl

Key
frequency
row percentage

Kan én eller fleres af disse ting hjælpe på din hovedpine? - At ligge ned	Kan én eller fleres af disse ting hjælpe på din hovedpine? - At sove		
	Nej	Ja	Total
Nej	37	84	121
	30.58	69.42	100.00
Ja	16	142	158
	10.13	89.87	100.00
Total	53	226	279
	19.00	81.00	100.00

```
.
. ** Dissing et al hence decided to define the migraine-tension-type index as the
. ** first PCA score based onthe severity and the symptom indices
.
. pca HAseverity IHAsymptoms

Principal components/correlation                 Number of obs    =       279
                                                 Number of comp.  =         2
                                                 Trace            =         2
    Rotation: (unrotated = principal)            Rho              =    1.0000

    --------------------------------------------------------------------------
       Component |   Eigenvalue   Difference         Proportion   Cumulative
    -------------+------------------------------------------------------------
           Comp1 |      1.45087      .901745             0.7254       0.7254
           Comp2 |      .549127            .             0.2746       1.0000
    --------------------------------------------------------------------------

Principal components (eigenvectors)

    ------------------------------------------------
        Variable |    Comp1     Comp2 | Unexplained
    -------------+--------------------+-------------
      HAseverity |   0.7071    0.7071 |           0
     IHAsymptoms |   0.7071   -0.7071 |           0
    ------------------------------------------------

. predict aux
(score assumed)
(1 components skipped)

Scoring coefficients
    sum of squares(column-loading) = 1

    ----------------------------------
        Variable |    Comp1     Comp2
    -------------+--------------------
      HAseverity |   0.7071
     IHAsymptoms |   0.7071   -0.7071
    ----------------------------------
```

```
. egen ImigraineTTH = std(aux)

. drop aux

. 
. *** For this paper, we decided to use besides the migraine-tension-type index
. *** also the single headache characteristics
. 
. 
. gen Ifrequency=frequency

. label define labfrequency 1 "1-2 days" 2 "3-5 days" 3 "nearly every day"

. label value Ifrequency labfrequency

. mytablist frequency Ifrequency

     +-----------------------------------------------+
     |       frequency_bl         Ifrequency   freq |
     |-----------------------------------------------|
  1. | 1 - 2 dage om ugen           1-2 days    125 |
  2. | 3 - 5 dage om ugen           3-5 days    116 |
  3. |    Næsten hver dag   nearly every day     38 |
     +-----------------------------------------------+

. 
. gen Iintensity = intensity

. 
. gen Iduration=duration

. label define labduration 1 "1/2-1 years" 2 "1-3 years" 3 ">3 years"

. label value Iduration labduration

. mytablist duration Iduration

     +-------------------------------------+
     |   duration_bl     Iduration   freq |
     |-------------------------------------|
  1. |      ½ - 1 år   1/2-1 years     64 |
  2. |      1 - 3 år     1-3 years    137 |
  3. | Mere end 3 år      >3 years     78 |
     +-------------------------------------+
```

```
.
. gen Ilength=length

. label define lablength 1 "<2 hours" 2 "2 hours - 1/2 day " 3 "the whole day" 4 "all day
> and night"

. label values Ilength lablength

. mytablist length Ilength

     +-----------------------------------------------------+
     |                   length             Ilength   freq |
     |-----------------------------------------------------|
  1. |       Mindre end 2 timer            <2 hours     32 |
  2. | Fra 2 timer op til ½ dag   2 hours - 1/2 day    189 |
  3. |               Hele dagen       the whole day     52 |
  4. |     Hele dagen og natten   all day and night      6 |
     +-----------------------------------------------------+

.
. gen Isickdays=sickdaysHA

. label define labsickdays 0 "0 days" 1 "1-5 days" 2 "5-20 days" 3 ">20 days"

. label values Isickdays labsickdays

. mytablist sickdaysHA Isickdays

     +------------------------------------------+
     |            sickdaysHA   Isickdays   freq |
     |------------------------------------------|
  1. |            0 sygedage      0 days     44 |
  2. |        1 - 5 sygedage    1-5 days    120 |
  3. |       5 - 20 sygedage   5-20 days     97 |
  4. | Flere end 20 sygedage    >20 days     18 |
     +------------------------------------------+

.
.
. ****************************************************************
. *** Sport activities ******************************************
```

```
. ****************************************************************
.
. ** The following variables provide information about sport activities:
.
. tab1 weeklysport_bl sports_*bl sportinjury_bl

-> tabulation of weeklysport_bl

Hvor mange gange |
 dyrker du sport |
   i løbet af en |
            uge? |      Freq.     Percent        Cum.
-----------------+-----------------------------------
         0 gange |         24        8.60        8.60
     1 - 3 gange |        194       69.53       78.14
Mere end 3 gange |         61       21.86      100.00
-----------------+-----------------------------------
           Total |        279      100.00

-> tabulation of sports_ballplay_bl

    Hvilken |
slags sport |
      eller |
     motion |
 dyrker du? |
 - Boldspil |      Freq.     Percent        Cum.
------------+-----------------------------------
        Nej |        109       42.75       42.75
         Ja |        146       57.25      100.00
------------+-----------------------------------
      Total |        255      100.00

-> tabulation of sports_running_bl

    Hvilken |
slags sport |
      eller |
     motion |
 dyrker du? |
      - Løb |      Freq.     Percent        Cum.
------------+-----------------------------------
        Nej |        199       78.04       78.04
         Ja |         56       21.96      100.00
------------+-----------------------------------
      Total |        255      100.00

-> tabulation of sports_atletics_bl
```

Hvilken slags sport eller motion dyrker du? - Anden atletik	Freq.	Percent	Cum.
Nej	235	92.16	92.16
Ja	20	7.84	100.00
Total	255	100.00	

-> tabulation of sports_riding_bl

Hvilken slags sport eller motion dyrker du? - Ridning	Freq.	Percent	Cum.
Nej	240	94.12	94.12
Ja	15	5.88	100.00
Total	255	100.00	

-> tabulation of sports_fitness_bl

Hvilken slags sport eller motion dyrker du? - Fitness	Freq.	Percent	Cum.
Nej	227	89.02	89.02
Ja	28	10.98	100.00
Total	255	100.00	

-> tabulation of sports_cycling_bl

Hvilken slags sport eller motion dyrker du? - Cykling	Freq.	Percent	Cum.
Nej	204	80.00	80.00
Ja	51	20.00	100.00
Total	255	100.00	

-> tabulation of sports_other_bl

Hvilken slags sport eller motion dyrker du? - Andet	Freq.	Percent	Cum.
Nej	126	49.41	49.41
Ja	129	50.59	100.00
Total	255	100.00	

-> tabulation of sportinjury_bl

Har du oplevet at være kommet til skade i leg eller sport og været nødt til at h	Freq.	Percent	Cum.
Nej	168	60.22	60.22
Ja, en enkelt gang	73	26.16	86.38
Ja, flere gange	38	13.62	100.00
Total	279	100.00	

.

```
.
. ** The number of sports is counted:
.
. egen numsports=rowtotal(sports_*bl)


.
. ** There is a reasonable degree of association among the variables:
.
. tab2 weeklysport_bl numsports sportinjury_bl , row

-> tabulation of weeklysport_bl by numsports

+----------------+
| Key            |
|----------------|
|   frequency    |
| row percentage |
+----------------+

Hvor mange gange |
 dyrker du sport |
   i løbet af en |                       numsports
            uge? |         0          1          2          3          4 |     Total
-----------------+-------------------------------------------------------+----------
         0 gange |        24          0          0          0          0 |        24
                 |    100.00       0.00       0.00       0.00       0.00 |    100.00
-----------------+-------------------------------------------------------+----------
     1 - 3 gange |         2        105         56         28          3 |       194
                 |      1.03      54.12      28.87      14.43       1.55 |    100.00
-----------------+-------------------------------------------------------+----------
Mere end 3 gange |         0         23         18          9          9 |        61
                 |      0.00      37.70      29.51      14.75      14.75 |    100.00
-----------------+-------------------------------------------------------+----------
           Total |        26        128         74         37         12 |       279
                 |      9.32      45.88      26.52      13.26       4.30 |    100.00


Hvor mange gange |
 dyrker du sport |
   i løbet af en | numsports
            uge? |         5 |     Total
-----------------+-----------+----------
         0 gange |         0 |        24
                 |      0.00 |    100.00
-----------------+-----------+----------
     1 - 3 gange |         0 |       194
                 |      0.00 |    100.00
-----------------+-----------+----------
Mere end 3 gange |         2 |        61
                 |      3.28 |    100.00
-----------------+-----------+----------
           Total |         2 |       279
                 |      0.72 |    100.00
```

-> tabulation of weeklysport_bl by sportinjury_bl

```
+-----------------+
| Key             |
|-----------------|
|   frequency     |
| row percentage  |
+-----------------+
```

Hvor mange gange dyrker du sport i løbet af en uge?	Har du oplevet at være kommet til skade i leg eller sport og været nødt til at h			
	Nej	Ja, en en	Ja, flere	Total
0 gange	17	6	1	24
	70.83	25.00	4.17	100.00
1 - 3 gange	126	43	25	194
	64.95	22.16	12.89	100.00
Mere end 3 gange	25	24	12	61
	40.98	39.34	19.67	100.00
Total	168	73	38	279
	60.22	26.16	13.62	100.00

-> tabulation of numsports by sportinjury_bl

```
+-----------------+
| Key             |
|-----------------|
|   frequency     |
| row percentage  |
+-----------------+
```

numsports	Har du oplevet at være kommet til skade i leg eller sport og været nødt til at h			
	Nej	Ja, en en	Ja, flere	Total
0	17	7	2	26
	65.38	26.92	7.69	100.00
1	84	29	15	128
	65.62	22.66	11.72	100.00
2	42	21	11	74
	56.76	28.38	14.86	100.00
3	21	10	6	37
	56.76	27.03	16.22	100.00

```
          4 |         3          6          3 |        12 
            |     25.00      50.00      25.00 |    100.00 
------------+---------------------------------+----------
          5 |         1          0          1 |         2 
            |     50.00       0.00      50.00 |    100.00 
------------+---------------------------------+----------
      Total |       168         73         38 |       279 
            |     60.22      26.16      13.62 |    100.00 

. pwcorr weeklysport_bl numsports sportinjury_bl

             | weekly~l numspo~s sporti~l
-------------+---------------------------
weeklyspor~l |   1.0000 
   numsports |   0.4795   1.0000 
sportinjur~l |   0.1875   0.1528   1.0000 


. 
. ** We decided to use directly the question on the weekly usage of sport:
. 
. gen Isport = weeklysport_bl

. label define labsport 0 "0 times" 1 "1-3 times" 2 ">3 times"

. label values Isport labsport


. 
. tab weeklysport_bl Isport

Hvor mange gange |
 dyrker du sport |
   i løbet af en |              Isport
            uge? |   0 times  1-3 times   >3 times |     Total
-----------------+---------------------------------+----------
         0 gange |        24          0          0 |        24 
     1 - 3 gange |         0        194          0 |       194 
Mere end 3 gange |         0          0         61 |        61 
-----------------+---------------------------------+----------
           Total |        24        194         61 |       279 


.
```

```
. local items `items' weeklysport_bl numsports sportinjury_bl

.
.
. ************************************************************************
. **** screen time and sleep time *****************************************
. ************************************************************************
.
. ** screentime and sleeptime depend on age:
.
. tab age screentime, row

+----------------+
| Key            |
|----------------|
|   frequency    |
| row percentage |
+----------------+

           | Hvor mange timer pr. dag bruger du omtrent
           |    på TV, computer, tablet og mobiltelef
     Alder | 0 - 1 tim  2 - 4 tim  5 - 6 tim  Mere end  |     Total
-----------+--------------------------------------------+----------
         7 |         6         17          0          0 |        23
           |     26.09      73.91       0.00       0.00 |    100.00
-----------+--------------------------------------------+----------
         8 |         5         24          1          0 |        30
           |     16.67      80.00       3.33       0.00 |    100.00
-----------+--------------------------------------------+----------
         9 |         4         26          0          1 |        31
           |     12.90      83.87       0.00       3.23 |    100.00
-----------+--------------------------------------------+----------
        10 |         4         34          1          1 |        40
           |     10.00      85.00       2.50       2.50 |    100.00
-----------+--------------------------------------------+----------
        11 |         0         37          7          1 |        45
           |      0.00      82.22      15.56       2.22 |    100.00
-----------+--------------------------------------------+----------
        12 |         0         28          7          7 |        42
           |      0.00      66.67      16.67      16.67 |    100.00
-----------+--------------------------------------------+----------
        13 |         2         23          8          5 |        38
           |      5.26      60.53      21.05      13.16 |    100.00
-----------+--------------------------------------------+----------
        14 |         2          6         13          9 |        30
           |      6.67      20.00      43.33      30.00 |    100.00
-----------+--------------------------------------------+----------
     Total |        23        195         37         24 |       279
           |      8.24      69.89      13.26       8.60 |    100.00
```

```
. tab age sleeptime, row

+-----------------+
| Key             |
|-----------------|
|    frequency    |
| row percentage  |
+-----------------+

           |  Hvor mange timer sover du pr.
           |             døgn?
     Alder | 6 - 8 tim  9 - 10 ti  11 - 12 t |     Total
-----------+---------------------------------+----------
         7 |         0         20          3 |        23 
           |      0.00      86.96      13.04 |    100.00 
-----------+---------------------------------+----------
         8 |         0         26          4 |        30 
           |      0.00      86.67      13.33 |    100.00 
-----------+---------------------------------+----------
         9 |         1         29          1 |        31 
           |      3.23      93.55       3.23 |    100.00 
-----------+---------------------------------+----------
        10 |         3         35          2 |        40 
           |      7.50      87.50       5.00 |    100.00 
-----------+---------------------------------+----------
        11 |         3         41          1 |        45 
           |      6.67      91.11       2.22 |    100.00 
-----------+---------------------------------+----------
        12 |        11         31          0 |        42 
           |     26.19      73.81       0.00 |    100.00 
-----------+---------------------------------+----------
        13 |        15         22          1 |        38 
           |     39.47      57.89       2.63 |    100.00 
-----------+---------------------------------+----------
        14 |        19         11          0 |        30 
           |     63.33      36.67       0.00 |    100.00 
-----------+---------------------------------+----------
     Total |        52        215         12 |       279 
           |     18.64      77.06       4.30 |    100.00 

.
```

```
. local items `items' screentime sleeptime

.
. ** There seem also to be slight effects of gender in the sense that girls
. ** tend to shorten the sleeping time earlier (reflecting the well know
. ** faster development of girls than boys?)
.
. bys sex: tab age screentime, row

-----------------------------------------------------------------------------------------
-> sex = Dreng

+----------------+
| Key            |
|----------------|
|   frequency    |
| row percentage |
+----------------+

           | Hvor mange timer pr. dag bruger du omtrent
           |    på TV, computer, tablet og mobiltelef
     Alder | 0 - 1 tim  2 - 4 tim  5 - 6 tim  Mere end  |     Total
-----------+--------------------------------------------+----------
         7 |         3          5          0          0 |         8
           |     37.50      62.50       0.00       0.00 |    100.00
-----------+--------------------------------------------+----------
         8 |         3         17          0          0 |        20
           |     15.00      85.00       0.00       0.00 |    100.00
-----------+--------------------------------------------+----------
         9 |         3          8          0          0 |        11
           |     27.27      72.73       0.00       0.00 |    100.00
-----------+--------------------------------------------+----------
        10 |         0         16          1          0 |        17
           |      0.00      94.12       5.88       0.00 |    100.00
-----------+--------------------------------------------+----------
        11 |         0         18          6          0 |        24
           |      0.00      75.00      25.00       0.00 |    100.00
-----------+--------------------------------------------+----------
        12 |         0         14          1          5 |        20
           |      0.00      70.00       5.00      25.00 |    100.00
-----------+--------------------------------------------+----------
        13 |         2         10          3          2 |        17
           |     11.76      58.82      17.65      11.76 |    100.00
-----------+--------------------------------------------+----------
        14 |         1          3          5          5 |        14
           |      7.14      21.43      35.71      35.71 |    100.00
-----------+--------------------------------------------+----------
     Total |        12         91         16         12 |       131
           |      9.16      69.47      12.21       9.16 |    100.00

-----------------------------------------------------------------------------------------
-> sex = Pige
```

```
+-----------------+
| Key             |
|-----------------|
|    frequency    |
| row percentage  |
+-----------------+
```

Alder	Hvor mange timer pr. dag bruger du omtrent på TV, computer, tablet og mobiltelef 0 - 1 tim	2 - 4 tim	5 - 6 tim	Mere end	Total
7	3	12	0	0	15
	20.00	80.00	0.00	0.00	100.00
8	2	7	1	0	10
	20.00	70.00	10.00	0.00	100.00
9	1	18	0	1	20
	5.00	90.00	0.00	5.00	100.00
10	4	18	0	1	23
	17.39	78.26	0.00	4.35	100.00
11	0	19	1	1	21
	0.00	90.48	4.76	4.76	100.00
12	0	14	6	2	22
	0.00	63.64	27.27	9.09	100.00
13	0	13	5	3	21
	0.00	61.90	23.81	14.29	100.00
14	1	3	8	4	16
	6.25	18.75	50.00	25.00	100.00
Total	11	104	21	12	148
	7.43	70.27	14.19	8.11	100.00

```
. bys sex: tab age sleeptime, row

-----------------------------------------------------------------------------------------
-> sex = Dreng

+-----------------+
| Key             |
|-----------------|
|    frequency    |
| row percentage  |
+-----------------+
```

Alder	Hvor mange timer sover du pr. døgn? 6 - 8 tim	9 - 10 ti	11 - 12 t	Total
7	0	7	1	8
	0.00	87.50	12.50	100.00
8	0	17	3	20
	0.00	85.00	15.00	100.00
9	0	11	0	11
	0.00	100.00	0.00	100.00
10	1	16	0	17
	5.88	94.12	0.00	100.00
11	2	22	0	24
	8.33	91.67	0.00	100.00
12	2	18	0	20
	10.00	90.00	0.00	100.00
13	3	14	0	17
	17.65	82.35	0.00	100.00
14	9	5	0	14
	64.29	35.71	0.00	100.00
Total	17	110	4	131
	12.98	83.97	3.05	100.00

-> sex = Pige

Key
frequency
row percentage

Alder	Hvor mange timer sover du pr. døgn? 6 - 8 tim	9 - 10 ti	11 - 12 t	Total
7	0	13	2	15
	0.00	86.67	13.33	100.00
8	0	9	1	10
	0.00	90.00	10.00	100.00
9	1	18	1	20
	5.00	90.00	5.00	100.00

```
-----------+--------------------------------+----------
        10 |         2         19          2 |        23 
           |      8.70      82.61       8.70 |    100.00 
-----------+--------------------------------+----------
        11 |         1         19          1 |        21 
           |      4.76      90.48       4.76 |    100.00 
-----------+--------------------------------+----------
        12 |         9         13          0 |        22 
           |     40.91      59.09       0.00 |    100.00 
-----------+--------------------------------+----------
        13 |        12          8          1 |        21 
           |     57.14      38.10       4.76 |    100.00 
-----------+--------------------------------+----------
        14 |        10          6          0 |        16 
           |     62.50      37.50       0.00 |    100.00 
-----------+--------------------------------+----------
     Total |        35        105          8 |       148 
           |     23.65      70.95       5.41 |    100.00 


. 
. ** In order to adjust for age, we use the following definitons. They lead to
. ** a relative high frquency of "normal" behaviour, as for most age groups
. ** the majority of children belong to one age group:
. 
. *sleeptime  short if 6-8 and age<=12
. *           long  if 11-12
. *                  normal oterhwise
. 
. *sreentime  short if 0-1  and 9<=age<=13 or 0-1 or 2-4 if age=14
. *           high if 5-6 or >6 and age<=12  or >6 and age >=13
. *                   long  otherhwise
. 
. gen Isleep = 2

. replace Isleep=1 if sleeptime==1 & age<=12
(18 real changes made)

. replace Isleep=3 if sleeptime==3
(12 real changes made)
```

```
. label define labsleep 1 "short" 2 "normal" 3 "long"

. label values Isleep labsleep

. tablist sleeptime age Isleep, s(v)

  +-----------------------------------------------------------------------+
  |  sleeptime_bl   age   Isleep   _Freq_   _Perc_   _CFreq_   _CPerc_ |
  |-----------------------------------------------------------------------|
  |   6 - 8 timer     9    short        1     0.36         1      0.36 |
  |   6 - 8 timer    10    short        3     1.08         4      1.43 |
  |   6 - 8 timer    11    short        3     1.08         7      2.51 |
  |   6 - 8 timer    12    short       11     3.94        18      6.45 |
  |   6 - 8 timer    13   normal       15     5.38        33     11.83 |
  |-----------------------------------------------------------------------|
  |   6 - 8 timer    14   normal       19     6.81        52     18.64 |
  |  9 - 10 timer     7   normal       20     7.17        72     25.81 |
  |  9 - 10 timer     8   normal       26     9.32        98     35.13 |
  |  9 - 10 timer     9   normal       29    10.39       127     45.52 |
  |  9 - 10 timer    10   normal       35    12.54       162     58.06 |
  |-----------------------------------------------------------------------|
  |  9 - 10 timer    11   normal       41    14.70       203     72.76 |
  |  9 - 10 timer    12   normal       31    11.11       234     83.87 |
  |  9 - 10 timer    13   normal       22     7.89       256     91.76 |
  |  9 - 10 timer    14   normal       11     3.94       267     95.70 |
  | 11 - 12 timer     7     long        3     1.08       270     96.77 |
  |-----------------------------------------------------------------------|
  | 11 - 12 timer     8     long        4     1.43       274     98.21 |
  | 11 - 12 timer     9     long        1     0.36       275     98.57 |
  | 11 - 12 timer    10     long        2     0.72       277     99.28 |
  | 11 - 12 timer    11     long        1     0.36       278     99.64 |
  | 11 - 12 timer    13     long        1     0.36       279    100.00 |
  +-----------------------------------------------------------------------+

.
. gen Iscreen=2

. replace Iscreen=1 if (screentime==1 & 9 <=age & age<=13) | (screentime<=2 & age==14)
(18 real changes made)

. replace Iscreen=3 if (screentime>=3 & age <=12 ) | (screentime==4 & age >=13)
(40 real changes made)
```

```
. label define labscreen 1 "short" 2 "normal" 3 "long"

. label values Iscreen labscreen

. tablist screentime age Iscreen, s(v)

  +-----------------------------------------------------------------------+
  |    screentime_bl   age   Iscreen   _Freq_   _Perc_   _CFreq_   _CPerc_ |
  |-----------------------------------------------------------------------|
  |       0 - 1 time     7    normal        6     2.15         6      2.15 |
  |       0 - 1 time     8    normal        5     1.79        11      3.94 |
  |       0 - 1 time     9     short        4     1.43        15      5.38 |
  |       0 - 1 time    10     short        4     1.43        19      6.81 |
  |       0 - 1 time    13     short        2     0.72        21      7.53 |
  |-----------------------------------------------------------------------|
  |       0 - 1 time    14     short        2     0.72        23      8.24 |
  |      2 - 4 timer     7    normal       17     6.09        40     14.34 |
  |      2 - 4 timer     8    normal       24     8.60        64     22.94 |
  |      2 - 4 timer     9    normal       26     9.32        90     32.26 |
  |      2 - 4 timer    10    normal       34    12.19       124     44.44 |
  |-----------------------------------------------------------------------|
  |      2 - 4 timer    11    normal       37    13.26       161     57.71 |
  |      2 - 4 timer    12    normal       28    10.04       189     67.74 |
  |      2 - 4 timer    13    normal       23     8.24       212     75.99 |
  |      2 - 4 timer    14     short        6     2.15       218     78.14 |
  |      5 - 6 timer     8      long        1     0.36       219     78.49 |
  |-----------------------------------------------------------------------|
  |      5 - 6 timer    10      long        1     0.36       220     78.85 |
  |      5 - 6 timer    11      long        7     2.51       227     81.36 |
  |      5 - 6 timer    12      long        7     2.51       234     83.87 |
  |      5 - 6 timer    13    normal        8     2.87       242     86.74 |
  |      5 - 6 timer    14    normal       13     4.66       255     91.40 |
  |-----------------------------------------------------------------------|
  | Mere end 6 timer     9      long        1     0.36       256     91.76 |
  | Mere end 6 timer    10      long        1     0.36       257     92.11 |
  | Mere end 6 timer    11      long        1     0.36       258     92.47 |
  | Mere end 6 timer    12      long        7     2.51       265     94.98 |
  | Mere end 6 timer    13      long        5     1.79       270     96.77 |
  |-----------------------------------------------------------------------|
  | Mere end 6 timer    14      long        9     3.23       279    100.00 |
  +-----------------------------------------------------------------------+
```

```
.
. tab1 Isleep Iscreen

-> tabulation of Isleep

     Isleep |      Freq.     Percent        Cum.
------------+-----------------------------------
      short |         18        6.45        6.45
     normal |        249       89.25       95.70
       long |         12        4.30      100.00
------------+-----------------------------------
      Total |        279      100.00

-> tabulation of Iscreen

    Iscreen |      Freq.     Percent        Cum.
------------+-----------------------------------
      short |         18        6.45        6.45
     normal |        221       79.21       85.66
       long |         40       14.34      100.00
------------+-----------------------------------
      Total |        279      100.00

.
. ** as a validation check, we compare sleep with sleepwell
.
. tab Isleep sleepwell , row

+----------------+
| Key            |
|----------------|
|   frequency    |
| row percentage |
+----------------+

           |   Plejer du at sove
           |         godt?
    Isleep |       Nej         Ja |     Total
-----------+----------------------+----------
     short |         5         13 |        18 
           |     27.78      72.22 |    100.00 
-----------+----------------------+----------
    normal |        18        231 |       249 
           |      7.23      92.77 |    100.00 
-----------+----------------------+----------
      long |         0         12 |        12 
           |      0.00     100.00 |    100.00 
-----------+----------------------+----------
     Total |        23        256 |       279 
           |      8.24      91.76 |    100.00 
```

```
.
.
. ***************************************************************************
. ***** Trauma intensity ****************************************************
. ***************************************************************************
.
. ** We first take a look at  the distribution of the variables of interest:
.
. tab1 hits_*bl  hit_*bl hospital_bl sportinjury_bl concussion_bl

-> tabulation of hits_notreat_bl

Hvor mange gange |
    har du slået |
 dit hoved eller |
  nakke, uden at |
         det var |
     nødvendigt  |      Freq.     Percent        Cum.
-----------------+-----------------------------------
         0 gange |         97       34.77       34.77
     1 - 3 gange |        121       43.37       78.14
Mere end 3 gange |         61       21.86      100.00
-----------------+-----------------------------------
           Total |        279      100.00

-> tabulation of hits_withtreat_bl

Hvor mange gange |
    har du slået |
 dit hoved eller |
  nakke, hvor du |
   har søgt læge |
           eller |      Freq.     Percent        Cum.
-----------------+-----------------------------------
         0 gange |        190       68.10       68.10
     1 - 3 gange |         79       28.32       96.42
Mere end 3 gange |         10        3.58      100.00
-----------------+-----------------------------------
           Total |        279      100.00

-> tabulation of hit_caracc_bl
```

Har du nogensinde været udsat for én eller flere af disse ting, hvor du har slåe	Freq.	Percent	Cum.
Nej	274	98.21	98.21
Ja	5	1.79	100.00
Total	279	100.00	

-> tabulation of hit_fallbike_bl

Har du nogensinde været udsat for én eller flere af disse ting, hvor du har slåe	Freq.	Percent	Cum.
Nej	220	78.85	78.85
Ja	59	21.15	100.00
Total	279	100.00	

-> tabulation of hit_fall2m_bl

Har du nogensinde været udsat for én eller flere af disse ting, hvor du har slåe	Freq.	Percent	Cum.
Nej	258	92.47	92.47
Ja	21	7.53	100.00
Total	279	100.00	

-> tabulation of hit_falltram_bl

Har du nogensinde været udsat for én eller flere af disse ting, hvor du har slåe	Freq.	Percent	Cum.
Nej	216	77.42	77.42
Ja	63	22.58	100.00
Total	279	100.00	

-> tabulation of hit_fallhorse_bl

Har du nogensinde været udsat for én eller flere af disse ting, hvor du har slåe	Freq.	Percent	Cum.
Nej	262	93.91	93.91
Ja	17	6.09	100.00
Total	279	100.00	

-> tabulation of hit_sports_bl

Har du nogensinde været udsat for én eller flere af disse ting, hvor du har slåe	Freq.	Percent	Cum.
Nej	160	57.35	57.35
Ja	119	42.65	100.00
Total	279	100.00	

-> tabulation of hit_violence_bl

Har du nogensinde været udsat for én eller flere af disse ting, hvor du har slåe	Freq.	Percent	Cum.
Nej	276	98.92	98.92
Ja	3	1.08	100.00
Total	279	100.00	

-> tabulation of hospital_bl

Har du været indlagt på hospitalet på grund af skade med hovedet eller nakken?	Freq.	Percent	Cum.
Nej	267	95.70	95.70
Ja	12	4.30	100.00
Total	279	100.00	

-> tabulation of sportinjury_bl

Har du oplevet at være kommet til skade i leg eller sport og været nødt til at h	Freq.	Percent	Cum.
Nej	168	60.22	60.22
Ja, en enkelt gang	73	26.16	86.38
Ja, flere gange	38	13.62	100.00
Total	279	100.00	

-> tabulation of concussion_bl

```
     Har du |
 nogensinde |
       haft |
hjerneryste |
       lse? |      Freq.     Percent        Cum.
------------+-----------------------------------
        Nej |        218       78.14       78.14
         Ja |         61       21.86      100.00
------------+-----------------------------------
      Total |        279      100.00

. 
. ** The hits_variables and hospital_bl tell us something about the traum intensity
. ** and it seems to be necessary in any case to merge this information into one
. ** variable. However, it is a little bit unclear whether we should just count
. ** traumata, or whether we should give those which required to
. ** see a doctor/to be hospitalized a higher weight. We consder two variants:
. 
. gen hitindex1 = hits_notreat_bl + 2 * hits_withtreat_bl + 3*hospital_bl

. gen hitindex2 = hits_notreat_bl + hits_withtreat_bl + hospital_bl


. 
. local items `items'  hits_notreat_bl  hits_withtreat_bl hospital_bl sportinjury_bl concu
> ssion_bl


. 
. tab1 hitindex*

-> tabulation of hitindex1

  hitindex1 |      Freq.     Percent        Cum.
------------+-----------------------------------
          0 |         78       27.96       27.96
          1 |         78       27.96       55.91
          2 |         48       17.20       73.12
          3 |         36       12.90       86.02
          4 |         21        7.53       93.55
          5 |          5        1.79       95.34
          6 |          8        2.87       98.21
          7 |          3        1.08       99.28
          8 |          1        0.36       99.64
          9 |          1        0.36      100.00
------------+-----------------------------------
      Total |        279      100.00

-> tabulation of hitindex2
```

```
  hitindex2 |      Freq.     Percent        Cum.
------------+-----------------------------------
          0 |         78       27.96       27.96
          1 |         95       34.05       62.01
          2 |         69       24.73       86.74
          3 |         28       10.04       96.77
          4 |          8        2.87       99.64
          5 |          1        0.36      100.00
------------+-----------------------------------
      Total |        279      100.00

.
.
. ** To have a further control variable, we also sum up the number of the
. ** specific hittypes recorded:
.
. egen numhit=rowtotal(hit_*bl)

. tablist hit_*bl numhit , s(v)

  +---------------------------------------------------------------------------------------+
  | hit_ca~l | hi~ke_bl | hi~2m_bl | hi~am_bl | hi~se_bl | hit_sp~l | hit_vi~l | numhit |
  |      Nej |      Nej |      Nej |      Nej |      Nej |      Nej |      Nej |      0 |
  |---------------------------------------------------------------------------------------|
  |       _Freq_        |        _Perc_        |        _CFreq_        |        _CPerc_        |
  |          118        |         42.29        |           118         |          42.29        |
  +---------------------------------------------------------------------------------------+

  +---------------------------------------------------------------------------------------+
  | hit_ca~l | hi~ke_bl | hi~2m_bl | hi~am_bl | hi~se_bl | hit_sp~l | hit_vi~l | numhit |
  |      Nej |      Nej |      Nej |      Nej |      Nej |       Ja |      Nej |      1 |
  |---------------------------------------------------------------------------------------|
  |       _Freq_        |        _Perc_        |        _CFreq_        |        _CPerc_        |
  |           44        |         15.77        |           162         |          58.06        |
  +---------------------------------------------------------------------------------------+

  +---------------------------------------------------------------------------------------+
  | hit_ca~l | hi~ke_bl | hi~2m_bl | hi~am_bl | hi~se_bl | hit_sp~l | hit_vi~l | numhit |
  |      Nej |      Nej |      Nej |      Nej |      Nej |       Ja |       Ja |      2 |
  |---------------------------------------------------------------------------------------|
  |       _Freq_        |        _Perc_        |        _CFreq_        |        _CPerc_        |
  |            1        |          0.36        |           163         |          58.42        |
  +---------------------------------------------------------------------------------------+

  +---------------------------------------------------------------------------------------+
  | hit_ca~l | hi~ke_bl | hi~2m_bl | hi~am_bl | hi~se_bl | hit_sp~l | hit_vi~l | numhit |
  |      Nej |      Nej |      Nej |      Nej |       Ja |      Nej |      Nej |      1 |
  |---------------------------------------------------------------------------------------|
  |       _Freq_        |        _Perc_        |        _CFreq_        |        _CPerc_        |
  |            4        |          1.43        |           167         |          59.86        |
  +---------------------------------------------------------------------------------------+
```

hit_ca~l	hi~ke_bl	hi~2m_bl	hi~am_bl	hi~se_bl	hit_sp~l	hit_vi~l	numhit
Nej	Nej	Nej	Nej	Ja	Ja	Nej	2

Freq	_Perc_	_CFreq_	_CPerc_
4	1.43	171	61.29

hit_ca~l	hi~ke_bl	hi~2m_bl	hi~am_bl	hi~se_bl	hit_sp~l	hit_vi~l	numhit
Nej	Nej	Nej	Ja	Nej	Nej	Nej	1

Freq	_Perc_	_CFreq_	_CPerc_
15	5.38	186	66.67

hit_ca~l	hi~ke_bl	hi~2m_bl	hi~am_bl	hi~se_bl	hit_sp~l	hit_vi~l	numhit
Nej	Nej	Nej	Ja	Nej	Ja	Nej	2

Freq	_Perc_	_CFreq_	_CPerc_
15	5.38	201	72.04

hit_ca~l	hi~ke_bl	hi~2m_bl	hi~am_bl	hi~se_bl	hit_sp~l	hit_vi~l	numhit
Nej	Nej	Nej	Ja	Nej	Ja	Ja	3

Freq	_Perc_	_CFreq_	_CPerc_
1	0.36	202	72.40

hit_ca~l	hi~ke_bl	hi~2m_bl	hi~am_bl	hi~se_bl	hit_sp~l	hit_vi~l	numhit
Nej	Nej	Ja	Nej	Nej	Nej	Nej	1

Freq	_Perc_	_CFreq_	_CPerc_
5	1.79	207	74.19

hit_ca~l	hi~ke_bl	hi~2m_bl	hi~am_bl	hi~se_bl	hit_sp~l	hit_vi~l	numhit
Nej	Nej	Ja	Nej	Nej	Ja	Nej	2

Freq	_Perc_	_CFreq_	_CPerc_
2	0.72	209	74.91

hit_ca~l	hi~ke_bl	hi~2m_bl	hi~am_bl	hi~se_bl	hit_sp~l	hit_vi~l	numhit
Nej	Nej	Ja	Ja	Nej	Nej	Nej	2

Freq	_Perc_	_CFreq_	_CPerc_
3	1.08	212	75.99

hit_ca~l	hi~ke_bl	hi~2m_bl	hi~am_bl	hi~se_bl	hit_sp~l	hit_vi~l	numhit
Nej	Nej	Ja	Ja	Nej	Ja	Nej	3

Freq	_Perc_	_CFreq_	_CPerc_
4	1.43	216	77.42

hit_ca~l	hi~ke_bl	hi~2m_bl	hi~am_bl	hi~se_bl	hit_sp~l	hit_vi~l	numhit
Nej	Ja	Nej	Nej	Nej	Nej	Nej	1

Freq	_Perc_	_CFreq_	_CPerc_
9	3.23	225	80.65

hit_ca~l	hi~ke_bl	hi~2m_bl	hi~am_bl	hi~se_bl	hit_sp~l	hit_vi~l	numhit
Nej	Ja	Nej	Nej	Nej	Ja	Nej	2

Freq	_Perc_	_CFreq_	_CPerc_
22	7.89	247	88.53

hit_ca~l	hi~ke_bl	hi~2m_bl	hi~am_bl	hi~se_bl	hit_sp~l	hit_vi~l	numhit
Nej	Ja	Nej	Nej	Ja	Ja	Nej	3

Freq	_Perc_	_CFreq_	_CPerc_
2	0.72	249	89.25

hit_ca~l	hi~ke_bl	hi~2m_bl	hi~am_bl	hi~se_bl	hit_sp~l	hit_vi~l	numhit
Nej	Ja	Nej	Ja	Nej	Nej	Nej	2

Freq	_Perc_	_CFreq_	_CPerc_
2	0.72	251	89.96

hit_ca~l	hi~ke_bl	hi~2m_bl	hi~am_bl	hi~se_bl	hit_sp~l	hit_vi~l	numhit
Nej	Ja	Nej	Ja	Nej	Ja	Nej	3

Freq	_Perc_	_CFreq_	_CPerc_
12	4.30	263	94.27

hit_ca~l	hi~ke_bl	hi~2m_bl	hi~am_bl	hi~se_bl	hit_sp~l	hit_vi~l	numhit
Nej	Ja	Nej	Ja	Nej	Ja	Ja	4

Freq	_Perc_	_CFreq_	_CPerc_
1	0.36	264	94.62

hit_ca~l	hi~ke_bl	hi~2m_bl	hi~am_bl	hi~se_bl	hit_sp~l	hit_vi~l	numhit
Nej	Ja	Nej	Ja	Ja	Nej	Nej	3

Freq	_Perc_	_CFreq_	_CPerc_
1	0.36	265	94.98

hit_ca~l	hi~ke_bl	hi~2m_bl	hi~am_bl	hi~se_bl	hit_sp~l	hit_vi~l	numhit
Nej	Ja	Nej	Ja	Ja	Ja	Nej	4

Freq	_Perc_	_CFreq_	_CPerc_
3	1.08	268	96.06

hit_ca~l	hi~ke_bl	hi~2m_bl	hi~am_bl	hi~se_bl	hit_sp~l	hit_vi~l	numhit
Nej	Ja	Ja	Nej	Nej	Nej	Nej	2

Freq	_Perc_	_CFreq_	_CPerc_
1	0.36	269	96.42

hit_ca~l	hi~ke_bl	hi~2m_bl	hi~am_bl	hi~se_bl	hit_sp~l	hit_vi~l	numhit
Nej	Ja	Ja	Ja	Nej	Ja	Nej	4

Freq	_Perc_	_CFreq_	_CPerc_
3	1.08	272	97.49

```
  +-------------------------------------------------------------------------------------+
  | hit_ca~l | hi~ke_bl | hi~2m_bl | hi~am_bl | hi~se_bl | hit_sp~l | hit_vi~l | numhit |
  |      Nej |       Ja |       Ja |       Ja |       Ja |       Ja |      Nej |      5 |
  |-------------------------------------------------------------------------------------|
  |       _Freq_        |        _Perc_        |        _CFreq_       |       _CPerc_      |
  |            2        |          0.72        |            274       |         98.21      |
  +-------------------------------------------------------------------------------------+

  +-------------------------------------------------------------------------------------+
  | hit_ca~l | hi~ke_bl | hi~2m_bl | hi~am_bl | hi~se_bl | hit_sp~l | hit_vi~l | numhit |
  |       Ja |      Nej |      Nej |      Nej |      Nej |      Nej |      Nej |      1 |
  |-------------------------------------------------------------------------------------|
  |       _Freq_        |        _Perc_        |        _CFreq_       |       _CPerc_      |
  |            2        |          0.72        |            276       |         98.92      |
  +-------------------------------------------------------------------------------------+

  +-------------------------------------------------------------------------------------+
  | hit_ca~l | hi~ke_bl | hi~2m_bl | hi~am_bl | hi~se_bl | hit_sp~l | hit_vi~l | numhit |
  |       Ja |      Nej |      Nej |      Nej |      Nej |       Ja |      Nej |      2 |
  |-------------------------------------------------------------------------------------|
  |       _Freq_        |        _Perc_        |        _CFreq_       |       _CPerc_      |
  |            2        |          0.72        |            278       |         99.64      |
  +-------------------------------------------------------------------------------------+

  +-------------------------------------------------------------------------------------+
  | hit_ca~l | hi~ke_bl | hi~2m_bl | hi~am_bl | hi~se_bl | hit_sp~l | hit_vi~l | numhit |
  |       Ja |       Ja |       Ja |       Ja |       Ja |       Ja |      Nej |      6 |
  |-------------------------------------------------------------------------------------|
  |       _Freq_        |        _Perc_        |        _CFreq_       |       _CPerc_      |
  |            1        |          0.36        |            279       |        100.00      |
  +-------------------------------------------------------------------------------------+

. 
. ** We then take a look at the association between the remaing candidate variables:
. ** There are distinct correlations. hitindex2 correlates stronger with numhit
. ** than hitindex1, which makes good sense, as hitindex1 is just "counting".
. ** hintindex1 correlates higher with concussions, which may indiate that it can
. ** make sense to take the severity of the traumata into account:
. 
. pwcorr hitindex* numhit sportinjury_bl concussion_bl

             | hitind~1 hitind~2   numhit sporti~l concus~l
-------------+---------------------------------------------
   hitindex1 |   1.0000 
   hitindex2 |   0.9522   1.0000 
      numhit |   0.3748   0.4569   1.0000 
sportinjur~l |   0.4063   0.3942   0.3309   1.0000 
concussion~l |   0.5399   0.4775   0.2279   0.3654   1.0000 
```

```
.
.
. * Since it is unclear whether it makes sense to combine the information on
. * trauma with the information om concussions, we define two separate candidate
. * variables:
.
. gen Itrauma = hitindex1

.
. gen Iconcussion = concussion_bl

. label define labyn 0 "no" 1 "yes"

. label values Iconcussion labyn

.
.
. ** We do not include sportinjury, as this reflects not only trauma intensity, but
. ** also sport activity.
.
.
.
. ******************************************************************************
. *** Neck pain history *************************************************
. ******************************************************************************
.
. *** There are two items on backpain/neckpain history:
.
. tab1 *pain_last*

-> tabulation of neckpain_lastyear_bl

 Har du det |
 seneste år |
haft ondt i |
    nakken? |      Freq.     Percent        Cum.
------------+-----------------------------------
        Nej |        117       41.94       41.94
         Ja |        162       58.06      100.00
------------+-----------------------------------
      Total |        279      100.00

-> tabulation of backpain_lastyear_bl
```

```
 Har du det |
 seneste år |
haft ondt i |
    ryggen? |      Freq.     Percent        Cum.
------------+-----------------------------------
        Nej |        159       56.99       56.99
         Ja |        120       43.01      100.00
------------+-----------------------------------
      Total |        279      100.00

.
. tab2 *pain_last* , row

-> tabulation of neckpain_lastyear_bl by backpain_lastyear_bl

+-----------------+
| Key             |
|-----------------|
|    frequency    |
| row percentage  |
+-----------------+

Har du det |
   seneste |  Har du det seneste
  år haft  |    år haft ondt i
   ondt i  |       ryggen?
  nakken?  |       Nej         Ja |     Total
-----------+----------------------+----------
       Nej |        92         25 |       117
           |     78.63      21.37 |    100.00
-----------+----------------------+----------
        Ja |        67         95 |       162
           |     41.36      58.64 |    100.00
-----------+----------------------+----------
     Total |       159        120 |       279
           |     56.99      43.01 |    100.00

.
. *** As there is no literature on an association between back pain and neck pain,
. *** only neck pain is defined as candidate variable. However, back pain is
. *** included in the item list.
```

```
.
. gen Ineck = neckpain_lastyear_bl

.
. label values Ineck labyn

. mytablist Ineck neckpain_lastyear_bl

     +--------------------------+
     | neckpa~l   Ineck   freq |
     |--------------------------|
  1. |      Nej      no    117 |
  2. |       Ja     yes    162 |
     +--------------------------+

.
. local items `items' backpain_lastyear_bl neckpain_lastyear_bl

.
.
. ***********************************************************************
. *** social/psychological environment ***********************************
. ***********************************************************************
.
. ** Direct information on the family income is only available for about half
. ** of the subjects, as this question was only asked at the followup within
. ** the randomized children:
.
. gen withincome=!mi(income)

. tab randomized withincome

           |      withincome
randomized |         0          1 |     Total
-----------+----------------------+----------
         0 |        80          0 |        80
         1 |        53        146 |       199
-----------+----------------------+----------
     Total |       133        146 |       279

.
```

```
. ** Besides sleep and screen there are some further variables, which might be
. ** indicators for the social/psychological environment
. ** prevtreat : previous treatment (indicating the interest of parents?)
. ** withcar : bringing the child by car to school
. ** smoking
. ** sports
.
.
. ** There are, however, no much signs that these variables are
. ** asscociated with income:
.
. gen withcar = transport_bl==4 if transport_bl!=5
(4 missing values generated)

.
. tab1 prevtreat_bl withcar smoking

-> tabulation of prevtreat_bl

     Har du |
  tidligere |
       fået |
 behandling |
    for din |
 hovedpine? |      Freq.     Percent        Cum.
------------+-----------------------------------
        Nej |        223       79.93       79.93
         Ja |         56       20.07      100.00
------------+-----------------------------------
      Total |        279      100.00

-> tabulation of withcar

    withcar |      Freq.     Percent        Cum.
------------+-----------------------------------
          0 |        193       70.18       70.18
          1 |         82       29.82      100.00
------------+-----------------------------------
      Total |        275      100.00

-> tabulation of smokingathome_bl

     Er der |
  nogen der |
      ryger |
 hjemme hos |
       jer? |      Freq.     Percent        Cum.
------------+-----------------------------------
        Nej |        230       82.44       82.44
         Ja |         49       17.56      100.00
------------+-----------------------------------
      Total |        279      100.00
```

```
.
. foreach var of varlist Isleep Iscreen weeklysport {
  2.   ologit `var' income
  3. }

Iteration 0:   log likelihood = -57.033692
Iteration 1:   log likelihood = -57.033439
Iteration 2:   log likelihood = -57.033439

Ordered logistic regression                             Number of obs =    146
                                                        LR chi2(1)    =   0.00
                                                        Prob > chi2   = 0.9820
Log likelihood = -57.033439                             Pseudo R2     = 0.0000

------------------------------------------------------------------------------
      Isleep | Coefficient  Std. err.      z    P>|z|     [95% conf. interval]
-------------+----------------------------------------------------------------
      income |   .0032142   .1427879     0.02   0.982     -.276645    .2830734
-------------+----------------------------------------------------------------
       /cut1 |  -2.488494   .8950366                     -4.242733   -.7342543
       /cut2 |   3.588452   .9821497                      1.663474     5.51343
------------------------------------------------------------------------------

Iteration 0:   log likelihood = -93.249608
Iteration 1:   log likelihood = -93.226796
Iteration 2:   log likelihood = -93.226791

Ordered logistic regression                             Number of obs =    146
                                                        LR chi2(1)    =   0.05
                                                        Prob > chi2   = 0.8308
Log likelihood = -93.226791                             Pseudo R2     = 0.0002

------------------------------------------------------------------------------
     Iscreen | Coefficient  Std. err.      z    P>|z|     [95% conf. interval]
-------------+----------------------------------------------------------------
      income |   .0225327   .1055922     0.21   0.831    -.1844241    .2294895
-------------+----------------------------------------------------------------
       /cut1 |  -2.478487   .6956586                     -3.841953   -1.115021
       /cut2 |   1.973647   .6710815                      .6583519    3.288943
------------------------------------------------------------------------------

Iteration 0:   log likelihood = -116.05989
Iteration 1:   log likelihood = -115.48653
Iteration 2:   log likelihood = -115.48507
Iteration 3:   log likelihood = -115.48507

Ordered logistic regression                             Number of obs =    146
                                                        LR chi2(1)    =   1.15
                                                        Prob > chi2   = 0.2836
Log likelihood = -115.48507                             Pseudo R2     = 0.0050
```

```
--------------------------------------------------------------------------------
weeklysport_bl | Coefficient  Std. err.      z    P>|z|     [95% conf. interval]
---------------+----------------------------------------------------------------
        income |   .1001894   .0941231     1.06   0.287    -.0842884    .2846672
---------------+----------------------------------------------------------------
         /cut1 |  -2.029023   .6283806                     -3.260626   -.7974194
         /cut2 |   1.686464   .6068503                      .4970597    2.875869
--------------------------------------------------------------------------------

.
. foreach var of varlist prevtreat_bl withcar smoking  {
  2.   logit `var' income
  3. }

Iteration 0:   log likelihood = -76.776395
Iteration 1:   log likelihood = -76.735631
Iteration 2:   log likelihood = -76.735616
Iteration 3:   log likelihood = -76.735616

Logistic regression                                     Number of obs =    146
                                                        LR chi2(1)    =   0.08
                                                        Prob > chi2   = 0.7752
Log likelihood = -76.735616                             Pseudo R2     = 0.0005

------------------------------------------------------------------------------
prevtreat_bl | Coefficient  Std. err.      z    P>|z|     [95% conf. interval]
-------------+----------------------------------------------------------------
      income |   -.029971   .1046209    -0.29   0.775    -.2350241    .1750822
       _cons |   -1.09462   .6426245    -1.70   0.089     -2.35414    .1649014
------------------------------------------------------------------------------

Iteration 0:   log likelihood = -82.352257
Iteration 1:   log likelihood = -80.821853
Iteration 2:   log likelihood = -80.810288
Iteration 3:   log likelihood = -80.810288

Logistic regression                                     Number of obs =    145
                                                        LR chi2(1)    =   3.08
                                                        Prob > chi2   = 0.0791
Log likelihood = -80.810288                             Pseudo R2     = 0.0187

------------------------------------------------------------------------------
     withcar | Coefficient  Std. err.      z    P>|z|     [95% conf. interval]
-------------+----------------------------------------------------------------
      income |  -.1734018   .0986028    -1.76   0.079    -.3666596    .0198561
       _cons |   -.076588   .5844419    -0.13   0.896    -1.222073    1.068897
------------------------------------------------------------------------------
```

```
Iteration 0:   log likelihood = -58.320401
Iteration 1:   log likelihood = -56.667685
Iteration 2:   log likelihood = -56.607973
Iteration 3:   log likelihood =  -56.60793
Iteration 4:   log likelihood =  -56.60793

Logistic regression                                     Number of obs =    146
                                                        LR chi2(1)    =   3.42
                                                        Prob > chi2   = 0.0642
Log likelihood = -56.60793                              Pseudo R2     = 0.0294

----------------------------------------------------------------------------------
smokingathome_bl | Coefficient  Std. err.      z    P>|z|     [95% conf. interval]
-----------------+----------------------------------------------------------------
          income |  -.2254941   .1210458    -1.86   0.062    -.4627395    .0117513
           _cons |  -.5781918   .6862392    -0.84   0.399    -1.923196    .7668123
----------------------------------------------------------------------------------

.
. ** Also the association among the variables is at least not very distinct
.
. pwcorr Isleep Iscreen prevtreat_bl withcar smoking income weeklysport

             |   Isleep  Iscreen pre~t_bl  withcar smokin~l   income weekly~l
-------------+---------------------------------------------------------------
      Isleep |   1.0000
     Iscreen |  -0.1836   1.0000
prevtreat_bl |  -0.0218  -0.0083   1.0000
     withcar |   0.0673  -0.0567  -0.0079   1.0000
smokingath~l |   0.0303  -0.0601  -0.0196   0.0208   1.0000
      income |   0.0032   0.0164  -0.0237  -0.1477  -0.1572   1.0000
weeklyspor~l |   0.0572  -0.0137   0.0096  -0.0761   0.0088   0.0996   1.0000

.
. tab Iscreen weeklysport, row

+----------------+
| Key            |
|----------------|
|   frequency    |
| row percentage |
+----------------+
```

```
           | Hvor mange gange dyrker du sport
           |        i løbet af en uge?
   Iscreen |   0 gange  1 - 3 gan  Mere end  |     Total
-----------+---------------------------------+----------
     short |         5          6          7 |        18 
           |     27.78      33.33      38.89 |    100.00 
-----------+---------------------------------+----------
    normal |        16        158         47 |       221 
           |      7.24      71.49      21.27 |    100.00 
-----------+---------------------------------+----------
      long |         3         30          7 |        40 
           |      7.50      75.00      17.50 |    100.00 
-----------+---------------------------------+----------
     Total |        24        194         61 |       279 
           |      8.60      69.53      21.86 |    100.00 

. 
. ** Hence we finally decided only to use the income as indicator
. 
. gen Iincome = income
(133 missing values generated)

. 
. label define labincome 1 "<`=round(200/7.45)'" 2 "`= round(250/7.45)' " ///
>   3 "`= round(350/7.45)' "  4 "`= round(450/7.45)' "   5 "`= round(550/7.45)' " ///
>   6 "`= round(650/7.45)' "  7 "`= round(750/7.45)' "   8 ">`= round(800/7.45)' "

. 
. label value Iincome labincome

. 
. tablist income Iincome

  +-----------------------------------------------------------------------+
  |                 income   Iincome   _Freq_   _Perc_   _CFreq_   _CPerc_ |
  |-----------------------------------------------------------------------|
  |                      .         .      133    47.67       133     47.67 |
  | Kr. 800.000 eller mere      >107       36    12.90       169     60.57 |
  |  Kr. 700.000 - 799.999       101       35    12.54       204     73.12 |
  |  Kr. 600.000 - 699.999        87       20     7.17       224     80.29 |
  |  Kr. 500.000 - 599.999        74       20     7.17       244     87.46 |
  |-----------------------------------------------------------------------|
  |  Kr. 400.000 - 499.999        60       14     5.02       258     92.47 |
  |  Kr. 300.000 - 399.999        47       12     4.30       270     96.77 |
  |  Kr. 200.000 - 299.999        34        7     2.51       277     99.28 |
  |      Under kr. 200.000       <27        2     0.72       279    100.00 |
  +-----------------------------------------------------------------------+
```

```
.
. *** The inccome is not included in the variable list for the exploratory part
. *** due to the occurrence of missing values
.
. *local items `items' income
.
. ****************************************************************
. **** Headache family *******************************************
. ****************************************************************
.
. *** There are three variables depicting this aspect:
.
. tab1 famhead*

-> tabulation of famheadache_mother_bl

Lider nogen |
      i din |
 familie af |
 hovedpine? |
      - Mor |      Freq.     Percent        Cum.
------------+-----------------------------------
        Nej |        127       45.52       45.52
         Ja |        152       54.48      100.00
------------+-----------------------------------
      Total |        279      100.00

-> tabulation of famheadache_father_bl

Lider nogen |
      i din |
 familie af |
 hovedpine? |
      - Far |      Freq.     Percent        Cum.
------------+-----------------------------------
        Nej |        208       74.55       74.55
         Ja |         71       25.45      100.00
------------+-----------------------------------
      Total |        279      100.00

-> tabulation of famheadache_sibling_bl

Lider nogen |
      i din |
 familie af |
 hovedpine? |
 - Søskende |      Freq.     Percent        Cum.
------------+-----------------------------------
        Nej |        240       86.02       86.02
         Ja |         39       13.98      100.00
------------+-----------------------------------
      Total |        279      100.00
```

```
.
. ** Interestingly, there is a negative association between
. ** mother and father:
.
. tab famheadache_m famheadache_f , row exact

+----------------+
| Key            |
|----------------|
|   frequency    |
| row percentage |
+----------------+

     Lider |
   nogen i |
       din |   Lider nogen i din
familie af | familie af hovedpine?
hovedpine? |         - Far
     - Mor |       Nej         Ja |     Total
-----------+----------------------+----------
       Nej |        81         46 |       127 
           |     63.78      36.22 |    100.00 
-----------+----------------------+----------
        Ja |       127         25 |       152 
           |     83.55      16.45 |    100.00 
-----------+----------------------+----------
     Total |       208         71 |       279 
           |     74.55      25.45 |    100.00 

           Fisher's exact =                 0.000
   1-sided Fisher's exact =                 0.000

.
. local items `items' famheadache_m famheadache_f famheadache_s

.
. ** We define a family index by summing up mother and father
.
. egen famhaindex = rowtotal(famheadache_m famheadache_f)

. tablist famheadache_m famheadache_f famhaindex, s(v)

  +-----------------------------------------------------------------------+
  | f~moth~l   f~fath~l   famhai~x   _Freq_   _Perc_   _CFreq_   _CPerc_ |
  |-----------------------------------------------------------------------|
  |      Nej        Nej          0       81    29.03        81     29.03 |
  |      Nej         Ja          1       46    16.49       127     45.52 |
  |       Ja        Nej          1      127    45.52       254     91.04 |
  |       Ja         Ja          2       25     8.96       279    100.00 |
  +-----------------------------------------------------------------------+
```

```
.
. ** There is a relation to headache in siblings:
.
. tab famhaindex famheadache_s, row

+----------------+
| Key            |
|----------------|
|   frequency    |
| row percentage |
+----------------+

           |   Lider nogen i din
           | familie af hovedpine?
           |      - Søskende
famhaindex |       Nej         Ja |     Total
-----------+----------------------+----------
         0 |        75          6 |        81 
           |     92.59       7.41 |    100.00 
-----------+----------------------+----------
         1 |       145         28 |       173 
           |     83.82      16.18 |    100.00 
-----------+----------------------+----------
         2 |        20          5 |        25 
           |     80.00      20.00 |    100.00 
-----------+----------------------+----------
     Total |       240         39 |       279 
           |     86.02      13.98 |    100.00 

. logit famheadache_s famhaindex

Iteration 0:   log likelihood = -112.87584  
Iteration 1:   log likelihood = -110.85415  
Iteration 2:   log likelihood = -110.82246  
Iteration 3:   log likelihood = -110.82245  

Logistic regression                                     Number of obs =    279
                                                        LR chi2(1)    =   4.11
                                                        Prob > chi2   = 0.0427
Log likelihood = -110.82245                             Pseudo R2     = 0.0182

-----------------------------------------------------------------------------------------
famheadache_sibling_bl | Coefficient  Std. err.      z    P>|z|     [95% conf. interval]
-----------------------+-----------------------------------------------------------------
            famhaindex |   .6053448   .3020349     2.00   0.045     .0133672    1.197322
                 _cons |  -2.345226   .3339216    -7.02   0.000      -2.9997   -1.690751
-----------------------------------------------------------------------------------------
```

```
.
. ** There is no relation to income:
.
. tab Iincome famhaindex, row

+----------------+
| Key            |
|----------------|
|   frequency    |
| row percentage |
+----------------+

           |             famhaindex
   Iincome |         0          1          2 |     Total
-----------+---------------------------------+----------
       <27 |         1          0          1 |         2
           |     50.00       0.00      50.00 |    100.00
-----------+---------------------------------+----------
      34   |         0          7          0 |         7
           |      0.00     100.00       0.00 |    100.00
-----------+---------------------------------+----------
      47   |         5          7          0 |        12
           |     41.67      58.33       0.00 |    100.00
-----------+---------------------------------+----------
      60   |         5          7          2 |        14
           |     35.71      50.00      14.29 |    100.00
-----------+---------------------------------+----------
      74   |         4         15          1 |        20
           |     20.00      75.00       5.00 |    100.00
-----------+---------------------------------+----------
      87   |         4         15          1 |        20
           |     20.00      75.00       5.00 |    100.00
-----------+---------------------------------+----------
     101   |        10         22          3 |        35
           |     28.57      62.86       8.57 |    100.00
-----------+---------------------------------+----------
    >107   |        14         18          4 |        36
           |     38.89      50.00      11.11 |    100.00
-----------+---------------------------------+----------
     Total |        43         91         12 |       146
           |     29.45      62.33       8.22 |    100.00
```

```
. ologit famhaindex Iincome

Iteration 0:   log likelihood = -125.56787
Iteration 1:   log likelihood = -125.41613
Iteration 2:   log likelihood = -125.41607
Iteration 3:   log likelihood = -125.41607

Ordered logistic regression                             Number of obs =    146
                                                        LR chi2(1)    =   0.30
                                                        Prob > chi2   = 0.5816
Log likelihood = -125.41607                             Pseudo R2     = 0.0012

------------------------------------------------------------------------------
  famhaindex | Coefficient  Std. err.      z    P>|z|     [95% conf. interval]
-------------+----------------------------------------------------------------
     Iincome |  -.0494403   .0898968    -0.55   0.582    -.2256348    .1267542
-------------+----------------------------------------------------------------
       /cut1 |  -1.165339   .5631663                     -2.269125   -.0615533
       /cut2 |   2.126506   .5984753                      .9535159    3.299496
------------------------------------------------------------------------------

.
. ** There is a weak associations to duration, sickdays, severity and migraineTTH:
.
. tab famhaindex frequency , row

+----------------+
| Key            |
|----------------|
|   frequency    |
| row percentage |
+----------------+

           |    Hvor tit har du hovedpine?
famhaindex | 1 - 2 dag  3 - 5 dag  Næsten hv |     Total
-----------+---------------------------------+----------
         0 |        28         37         16 |        81 
           |     34.57      45.68      19.75 |    100.00 
-----------+---------------------------------+----------
         1 |        88         67         18 |       173 
           |     50.87      38.73      10.40 |    100.00 
-----------+---------------------------------+----------
         2 |         9         12          4 |        25 
           |     36.00      48.00      16.00 |    100.00 
-----------+---------------------------------+----------
     Total |       125        116         38 |       279 
           |     44.80      41.58      13.62 |    100.00 
```

```
. tab famhaindex duration , row

+-----------------+
| Key             |
|-----------------|
|   frequency     |
| row percentage  |
+-----------------+

           |      Hvor længe har du haft
           |            hovedpine?
famhaindex |  ½ - 1 år   1 - 3 år  Mere end  |     Total
-----------+---------------------------------+----------
         0 |        25         41         15 |        81
           |     30.86      50.62      18.52 |    100.00
-----------+---------------------------------+----------
         1 |        36         81         56 |       173
           |     20.81      46.82      32.37 |    100.00
-----------+---------------------------------+----------
         2 |         3         15          7 |        25
           |     12.00      60.00      28.00 |    100.00
-----------+---------------------------------+----------
     Total |        64        137         78 |       279
           |     22.94      49.10      27.96 |    100.00

. tabstat Iintensity, by(famhaindex)

Summary for variables: Iintensity
Group variable: famhaindex

famhaindex |      Mean
-----------+----------
         0 |  5.802469
         1 |  6.075145
         2 |       5.8
-----------+----------
     Total |  5.971326
----------------------

. tab famhaindex sickdays , row

+-----------------+
| Key             |
|-----------------|
|   frequency     |
| row percentage  |
+-----------------+
```

famhaindex	Hvor mange sygedage har du haft pga. hovedpine det seneste år? 0 sygedag	1 - 5 syg	5 - 20 sy	Flere end	Total
0	14	43	19	5	81
	17.28	53.09	23.46	6.17	100.00
1	25	70	68	10	173
	14.45	40.46	39.31	5.78	100.00
2	5	7	10	3	25
	20.00	28.00	40.00	12.00	100.00
Total	44	120	97	18	279
	15.77	43.01	34.77	6.45	100.00

```
. tab famhaindex length , row

+----------------+
| Key            |
|----------------|
|   frequency    |
| row percentage |
+----------------+
```

famhaindex	Hvor lang tid varer din hovedpine typisk? Mindre en	Fra 2 tim	Hele dage	Hele dage	Total
0	10	56	14	1	81
	12.35	69.14	17.28	1.23	100.00
1	20	116	34	3	173
	11.56	67.05	19.65	1.73	100.00
2	2	17	4	2	25
	8.00	68.00	16.00	8.00	100.00
Total	32	189	52	6	279
	11.47	67.74	18.64	2.15	100.00

```
. tab famhaindex medication , row

+----------------+
| Key            |
|----------------|
|   frequency    |
| row percentage |
+----------------+
```

```
           |                  medication
famhaindex |         1          2          3          4 |     Total
-----------+--------------------------------------------+----------
         0 |         8         48         23          2 |        81
           |      9.88      59.26      28.40       2.47 |    100.00
-----------+--------------------------------------------+----------
         1 |        21         96         48          8 |       173
           |     12.14      55.49      27.75       4.62 |    100.00
-----------+--------------------------------------------+----------
         2 |         3         12          8          2 |        25
           |     12.00      48.00      32.00       8.00 |    100.00
-----------+--------------------------------------------+----------
     Total |        32        156         79         12 |       279
           |     11.47      55.91      28.32       4.30 |    100.00

.
. pwcorr famhaindex frequency duration Iintensity sickdays length medication ///
>    IHA* ImigraineTTH age

             | famhai~x freque~l durati~l Iinten~y sickda~A   length medica~n
-------------+---------------------------------------------------------------
  famhaindex |   1.0000
frequency_bl |  -0.0922   1.0000
 duration_bl |   0.1453   0.0026   1.0000
  Iintensity |   0.0353  -0.0925   0.1643   1.0000
  sickdaysHA |   0.1048   0.0616   0.1891   0.2545   1.0000
      length |   0.0646   0.1090   0.0774   0.1322   0.2291   1.0000
  medication |   0.0368   0.0155   0.1519   0.1326   0.1696   0.0730   1.0000
 IHAsymptoms |   0.0499  -0.1273   0.3163   0.3937   0.2373   0.0055   0.1620
ImigraineTTH |   0.1061  -0.0775   0.5344   0.6087   0.5428   0.1287   0.4150
         age |  -0.0240   0.2025   0.1188   0.0174   0.1715   0.0963   0.2395

             | IHAsym~s Imigra~H      age
-------------+---------------------------
 IHAsymptoms |   1.0000
ImigraineTTH |   0.8517   1.0000
         age |  -0.0565   0.0930   1.0000

.
. ologit frequency famhaindex

Iteration 0:   log likelihood = -277.92413
Iteration 1:   log likelihood = -276.80493
Iteration 2:   log likelihood = -276.80425
Iteration 3:   log likelihood = -276.80425

Ordered logistic regression                       Number of obs =    279
                                                  LR chi2(1)    =   2.24
                                                  Prob > chi2   = 0.1345
Log likelihood = -276.80425                       Pseudo R2     = 0.0040
```

```
------------------------------------------------------------------------------
frequency_bl | Coefficient  Std. err.      z    P>|z|     [95% conf. interval]
-------------+----------------------------------------------------------------
  famhaindex |  -.2927035   .1963705    -1.49   0.136    -.6775825    .0921756
-------------+----------------------------------------------------------------
       /cut1 |  -.4451859   .1991891                     -.8355895   -.0547824
       /cut2 |    1.62335   .2278254                       1.17682    2.069879
------------------------------------------------------------------------------

. ologit duration famhaindex

Iteration 0:   log likelihood = -291.07889
Iteration 1:   log likelihood =  -288.2401
Iteration 2:   log likelihood =   -288.234
Iteration 3:   log likelihood =   -288.234

Ordered logistic regression                             Number of obs =    279
                                                        LR chi2(1)    =   5.69
                                                        Prob > chi2   = 0.0171
Log likelihood = -288.234                               Pseudo R2     = 0.0098

------------------------------------------------------------------------------
 duration_bl | Coefficient  Std. err.      z    P>|z|     [95% conf. interval]
-------------+----------------------------------------------------------------
  famhaindex |   .4570387   .1927371     2.37   0.018     .0792808    .8347965
-------------+----------------------------------------------------------------
       /cut1 |  -.8640111   .2018217                     -1.259574   -.4684479
       /cut2 |   1.330165   .2124824                       .913707    1.746623
------------------------------------------------------------------------------

. ologit Iintensity famhaindex

Iteration 0:   log likelihood = -528.54951
Iteration 1:   log likelihood = -528.48057
Iteration 2:   log likelihood = -528.48057

Ordered logistic regression                             Number of obs =    279
                                                        LR chi2(1)    =   0.14
                                                        Prob > chi2   = 0.7104
Log likelihood = -528.48057                             Pseudo R2     = 0.0001

------------------------------------------------------------------------------
  Iintensity | Coefficient  Std. err.      z    P>|z|     [95% conf. interval]
-------------+----------------------------------------------------------------
  famhaindex |     .06623   .1784013     0.37   0.710    -.2834301    .4158902
-------------+----------------------------------------------------------------
       /cut1 |  -4.878812   .7232308                     -6.296319   -3.461306
       /cut2 |  -2.508359   .2717576                     -3.040994   -1.975724
       /cut3 |  -1.444275   .2107708                     -1.857379   -1.031172
       /cut4 |  -.4061486   .1890377                     -.7766556   -.0356416
       /cut5 |   .5737048   .1897483                      .201805    .9456047
       /cut6 |   1.701498   .2165488                       1.27707    2.125925
       /cut7 |   2.788195   .2885089                      2.222728    3.353662
```

```
       /cut8 |   3.713452   .4092914                      2.911256    4.515649
------------------------------------------------------------------------------

. ologit sickdays famhaindex,

Iteration 0:   log likelihood = -334.33108
Iteration 1:   log likelihood = -332.39637
Iteration 2:   log likelihood = -332.39404
Iteration 3:   log likelihood = -332.39404

Ordered logistic regression                             Number of obs =    279
                                                        LR chi2(1)    =   3.87
                                                        Prob > chi2   = 0.0490
Log likelihood = -332.39404                             Pseudo R2     = 0.0058

------------------------------------------------------------------------------
  sickdaysHA | Coefficient  Std. err.      z    P>|z|     [95% conf. interval]
-------------+----------------------------------------------------------------
  famhaindex |   .3820144   .1947679     1.96   0.050     .0002764    .7637523
-------------+----------------------------------------------------------------
       /cut1 |  -1.394333   .2156387                     -1.816977   -.9716885
       /cut2 |   .6582249   .1977959                       .270552    1.045898
       /cut3 |   3.000877   .2980245                       2.41676    3.584995
------------------------------------------------------------------------------

. ologit length famhaindex

Iteration 0:   log likelihood = -253.29912
Iteration 1:   log likelihood = -252.92713
Iteration 2:   log likelihood =  -252.9268
Iteration 3:   log likelihood =  -252.9268

Ordered logistic regression                             Number of obs =    279
                                                        LR chi2(1)    =   0.74
                                                        Prob > chi2   = 0.3882
Log likelihood = -252.9268                              Pseudo R2     = 0.0015

------------------------------------------------------------------------------
      length | Coefficient  Std. err.      z    P>|z|     [95% conf. interval]
-------------+----------------------------------------------------------------
  famhaindex |   .1867277   .2165169     0.86   0.388    -.2376375     .611093
-------------+----------------------------------------------------------------
       /cut1 |  -1.899113   .2496273                     -2.388374   -1.409853
       /cut2 |   1.489031   .2315875                      1.035128    1.942934
       /cut3 |   3.971336   .4510109                      3.087371    4.855301
------------------------------------------------------------------------------
```

```
. ologit medication famhaindex

Iteration 0:   log likelihood = -297.42174
Iteration 1:   log likelihood = -297.29009
Iteration 2:   log likelihood = -297.29007

Ordered logistic regression                             Number of obs =    279
                                                        LR chi2(1)    =   0.26
                                                        Prob > chi2   = 0.6078
Log likelihood = -297.29007                             Pseudo R2     = 0.0004

------------------------------------------------------------------------------
  medication | Coefficient  Std. err.      z    P>|z|     [95% conf. interval]
-------------+----------------------------------------------------------------
  famhaindex |   .1021831   .1991462     0.51   0.608    -.2881362    .4925024
-------------+----------------------------------------------------------------
       /cut1 |   -1.96431   .2425149                      -2.43963   -1.488989
       /cut2 |   .8062471   .2032339                       .407916    1.204578
       /cut3 |   3.184899   .3368882                       2.52461    3.845188
------------------------------------------------------------------------------

.
.
. regress HAseverity famhaindex

      Source |       SS           df       MS      Number of obs   =       279
-------------+----------------------------------   F(1, 277)       =      4.83
       Model |   4.7649327         1   4.7649327   Prob > F        =    0.0288
    Residual |  273.235068       277  .986408187   R-squared       =    0.0171
-------------+----------------------------------   Adj R-squared   =    0.0136
       Total |         278       278           1   Root MSE        =    .99318

------------------------------------------------------------------------------
  HAseverity | Coefficient  Std. err.      t    P>|t|     [95% conf. interval]
-------------+----------------------------------------------------------------
  famhaindex |   .2242416   .1020272     2.20   0.029     .0233943    .4250889
       _cons |  -.1792325   .1009242    -1.78   0.077    -.3779084    .0194433
------------------------------------------------------------------------------

. regress HAtime  famhaindex

      Source |       SS           df       MS      Number of obs   =       279
-------------+----------------------------------   F(1, 277)       =      0.18
       Model |  .172192817         1  .172192817   Prob > F        =    0.6723
    Residual |  265.985513       277   .96023651   R-squared       =    0.0006
-------------+----------------------------------   Adj R-squared   =   -0.0030
       Total |  266.157706       278   .95740182   Root MSE        =    .97992
```

```
------------------------------------------------------------------------------
      HAtime | Coefficient  Std. err.      t    P>|t|     [95% conf. interval]
-------------+----------------------------------------------------------------
  famhaindex |   -.042628   .1006646    -0.42   0.672    -.2407929    .1555368
       _cons |   1.836939   .0995763    18.45   0.000     1.640917    2.032962
------------------------------------------------------------------------------

. regress IHAsymptoms famhaindex

      Source |       SS           df       MS      Number of obs   =       279
-------------+----------------------------------   F(1, 277)       =      0.69
       Model |  1.13701008         1  1.13701008   Prob > F        =    0.4067
    Residual |  456.052954       277  1.64640056   R-squared       =    0.0025
-------------+----------------------------------   Adj R-squared   =   -0.0011
       Total |  457.189964       278  1.64456822   Root MSE        =    1.2831

------------------------------------------------------------------------------
 IHAsymptoms | Coefficient  Std. err.      t    P>|t|     [95% conf. interval]
-------------+----------------------------------------------------------------
  famhaindex |   .1095393   .1318122     0.83   0.407    -.1499416    .3690202
       _cons |   1.812089   .1303871    13.90   0.000     1.555413    2.068764
------------------------------------------------------------------------------

. regress ImigraineTTH famhaindex

      Source |       SS           df       MS      Number of obs   =       279
-------------+----------------------------------   F(1, 277)       =      3.16
       Model |   3.1313472         1   3.1313472   Prob > F        =    0.0768
    Residual |  274.868653       277  .992305607   R-squared       =    0.0113
-------------+----------------------------------   Adj R-squared   =    0.0077
       Total |         278       278           1   Root MSE        =    .99615

------------------------------------------------------------------------------
ImigraineTTH | Coefficient  Std. err.      t    P>|t|     [95% conf. interval]
-------------+----------------------------------------------------------------
  famhaindex |   .1817831   .1023318     1.78   0.077    -.0196637    .3832298
       _cons |  -.1452961   .1012255    -1.44   0.152     -.344565    .0539728
------------------------------------------------------------------------------

.
. rename famhaindex Ifamily
```

```
. label define labfamily 0 "none" 1 "one parent" 2 "both parents"

. label values Ifamily labfamily

. tablist famheadache_m famheadache_f Ifamily

  +-------------------------------------------------------------------------+
  | f~moth~l   f~fath~l        Ifamily   _Freq_   _Perc_   _CFreq_   _CPerc_ |
  |-------------------------------------------------------------------------|
  |       Ja        Nej     one parent      127    45.52       127     45.52 |
  |      Nej        Nej           none       81    29.03       208     74.55 |
  |      Nej         Ja     one parent       46    16.49       254     91.04 |
  |       Ja         Ja   both parents       25     8.96       279    100.00 |
  +-------------------------------------------------------------------------+

.
. ****************************************************************************
. *** Expected benefit index         ****************************************
. ****************************************************************************
.
.
.
.
. gen auxage = 0.5*((Iage <=9) + (Iage<=12))

. mytablist Iage auxage

     +----------------------+
     | Iage   auxage   freq |
     |----------------------|
  1. |    7        1     23 |
  2. |    8        1     30 |
  3. |    9        1     31 |
  4. |   10       .5     40 |
  5. |   11       .5     45 |
     |----------------------|
  6. |   12       .5     42 |
  7. |   13        0     38 |
  8. |   14        0     30 |
     +----------------------+
```

```
. gen auxfreq = Ifrequency!=3

. mytablist Ifrequency auxfreq

     +------------------------------------+
     |       Ifrequency   auxfreq   freq |
     |------------------------------------|
  1. |         1-2 days         1    125 |
  2. |         3-5 days         1    116 |
  3. | nearly every day         0     38 |
     +------------------------------------+

. gen auxscreen = (Iscreen<=2)

. tab Iscreen auxscreen

           |       auxscreen
   Iscreen |         0          1 |     Total
-----------+----------------------+----------
     short |         0         18 |        18
    normal |         0        221 |       221
      long |        40          0 |        40
-----------+----------------------+----------
     Total |        40        239 |       279

. gen auxsport =  Isport!=0

. mytablist weeklysport_bl Isport auxsport

     +-----------------------------------------------+
     |   weeklysport_bl      Isport   auxsport   freq |
     |-----------------------------------------------|
  1. |          0 gange     0 times          0     24 |
  2. |      1 - 3 gange   1-3 times          1    194 |
  3. | Mere end 3 gange    >3 times          1     61 |
     +-----------------------------------------------+

. gen auxtrauma =  0.5* ( (Itrauma>1)  + (Itrauma>0) )

. tab Itrauma auxtrauma

           |            auxtrauma
   Itrauma |         0         .5          1 |     Total
-----------+---------------------------------+----------
         0 |        78          0          0 |        78
         1 |         0         78          0 |        78
         2 |         0          0         48 |        48
         3 |         0          0         36 |        36
         4 |         0          0         21 |        21
         5 |         0          0          5 |         5
         6 |         0          0          8 |         8
         7 |         0          0          3 |         3
         8 |         0          0          1 |         1
```

```
         9 |          0          0          1 |          1
-----------+---------------------------------+----------
     Total |         78         78        123 |        279

. egen  Iexpected = rowtotal(auxage-auxtrauma)

.
. mytablist Iexpected auxage-auxtrauma
```

	auxage	auxfreq	auxscr~n	auxsport	auxtra~a	Iexpec~d	freq
1.	0	0	0	1	0	1	1
2.	0	0	1	0	0	1	2
3.	0	0	0	1	.5	1.5	1
4.	0	0	1	0	.5	1.5	2
5.	.5	0	0	1	0	1.5	1
6.	.5	1	0	0	0	1.5	1
7.	1	0	0	0	.5	1.5	1
8.	0	0	0	1	1	2	2
9.	0	0	1	0	1	2	2
10.	0	0	1	1	0	2	2
11.	0	1	0	1	0	2	2
12.	0	1	1	0	0	2	1
13.	.5	0	0	1	.5	2	3
14.	.5	1	0	0	.5	2	1
15.	0	0	1	1	.5	2.5	4
16.	0	1	0	1	.5	2.5	4
17.	.5	0	1	1	0	2.5	2
18.	.5	1	0	1	0	2.5	5
19.	.5	1	1	0	0	2.5	3
20.	1	0	1	0	.5	2.5	1
21.	0	0	1	1	1	3	3
22.	0	1	0	1	1	3	4
23.	0	1	1	0	1	3	4
24.	0	1	1	1	0	3	6
25.	.5	0	1	1	.5	3	2
26.	.5	1	0	1	.5	3	5
27.	.5	1	1	0	.5	3	1
28.	1	0	1	1	0	3	3
29.	1	1	1	0	0	3	2
30.	0	1	1	1	.5	3.5	9
31.	.5	0	1	1	1	3.5	3
32.	.5	1	0	1	1	3.5	8
33.	.5	1	1	0	1	3.5	1
34.	.5	1	1	1	0	3.5	27
35.	1	0	1	1	.5	3.5	1

```
     |--------------------------------------------------------------------|
 36. |     1         1          1          1         .5          3.5       1 |
```

```
     |--------------------------------------------------------------------|
 36. |     1         1          1          0         .5          3.5       1 |
 37. |     0         1          1          1          1            4      19 |
 38. |    .5         1          1          1         .5            4      20 |
 39. |     1         0          1          1          1            4       2 |
 40. |     1         1          0          1          1            4       1 |
     |--------------------------------------------------------------------|
 41. |     1         1          1          0          1            4       1 |
 42. |     1         1          1          1          0            4      20 |
 43. |    .5         1          1          1          1          4.5      44 |
 44. |     1         1          1          1         .5          4.5      22 |
 45. |     1         1          1          1          1            5      29 |
     +--------------------------------------------------------------------+

.
. pwcorr aux* Iexpected

             |   auxage  auxfreq auxscr~n auxsport auxtra~a Iexpec~d
-------------+------------------------------------------------------
      auxage |   1.0000
     auxfreq |   0.1871   1.0000
   auxscreen |   0.1987   0.1059   1.0000
    auxsport |   0.1108   0.1763  -0.0161   1.0000
   auxtrauma |  -0.0852   0.0643   0.0178   0.0747   1.0000
   Iexpected |   0.5490   0.5798   0.5109   0.4494   0.4797   1.0000

.
. label var Iintensity "Intensity of headache"

. label var Ifrequency "Frequency of headache"

. label var Iduration "Duration of headache"

. label var Ilength "Length of episodes"

. label var Isickdays "Absence from school"

. label var IHAsymptoms "Co-occuring symptoms"

. label var ImigraineTTH "Migraine-TTH"
```

```
. label var Iage "Age"

. label var Isport "Sport activity"

. label var Iscreen "Screen time"

. label var Isleep "Sleep duration"

. label var Itrauma "Trauma experience"

. label var Iconcuss "History of concussions"

. label var Ineck "History of neck pain"

. label var Iincome "Socioeconomic status"

. label var Ifamily "Headache in the family"

. label var Iexpected "Expected benefit index"

.
.
. log close
      name:  <unnamed>
       log:  /Users/basler-akademie/work/nikkb/hovedpine/finaldata/hpmodify/data/prepare.l
> og
  log type:  text
 closed on:   2 Feb 2023, 17:20:05
------------------------------------------------------------------------------------------
```
