# Supplementary material for: Potential effect modifiers for treatment with chiropractic manipulation versus sham manipulation for recurrent headaches in children aged 7–14 years: development of and results from a secondary analysis of a randomised clinical trial
Source: Chiropr Man Therap. 2023 Jul 11;31:20. doi: 10.1186/s12998-023-00492-2 (PMC10337090; doi:10.1186/s12998-023-00492-2)
Supplement: Supplementary file 3 — Additional file 3. Supplementary Figure 1. The pairwise correlations among the 17 candidate variables. [file 12998_2023_492_MOESM3_ESM.docx]

**Supplementary Figures**

Supplementary Figure 1: The pairwise correlations among the 17 candidate variables
